# Circulating small extracellular vesicles in Alzheimer’s disease: a case–control study of neuro-inflammation and synaptic dysfunction

**DOI:** 10.1186/s12916-024-03475-z

**Published:** 2024-06-20

**Authors:** Rishabh Singh, Sanskriti Rai, Prahalad Singh Bharti, Sadaqa Zehra, Priya Kumari Gorai, Gyan Prakash Modi, Neerja Rani, Kapil Dev, Krishna Kishore Inampudi, Vishnu V. Y., Prasun Chatterjee, Fredrik Nikolajeff, Saroj Kumar

**Affiliations:** 1 Department of Biophysics, All India Institute of Medical Sciences, New Delhi, 110029 India; 2 Department of Anatomy, All India Institute of Medical Sciences, New Delhi, India; 3 Department of Pharmaceutical Engineering and Technology, Indian Institute of Technology BHU, Varanasi, India; 4 Department of Biotechnology, Jamia Millia Islamia, New Delhi, India; 5 Department of Neurology, All India Institute of Medical Sciences, New Delhi, India; 6 Department of Geriatric Medicine, All India Institute of Medical Sciences, New Delhi, India; 7 Department of Health, Education, and Technology, Lulea University of Technology, Lulea, 97187 Sweden

**Keywords:** Alzheimer’s disease, Mild cognitive impairment, Small extracellular vesicles, Synaptic dysfunction, Neuroinflammation

## Abstract

**Background:**

Alzheimer’s disease (AD) is a neurodegenerative disease characterized by Aβ plaques and neurofibrillary tangles. Chronic inflammation and synaptic dysfunction lead to disease progression and cognitive decline. Small extracellular vesicles (sEVs) are implicated in AD progression by facilitating the spread of pathological proteins and inflammatory cytokines. This study investigates synaptic dysfunction and neuroinflammation protein markers in plasma-derived sEVs (PsEVs), their association with Amyloid-β and tau pathologies, and their correlation with AD progression.

**Methods:**

A total of 90 [AD = 35, mild cognitive impairment (MCI) = 25, and healthy age-matched controls (AMC) = 30] participants were recruited. PsEVs were isolated using a chemical precipitation method, and their morphology was characterized by transmission electron microscopy. Using nanoparticle tracking analysis, the size and concentration of PsEVs were determined. Antibody-based validation of PsEVs was done using CD63, CD81, TSG101, and L1CAM antibodies. Synaptic dysfunction and neuroinflammation were evaluated with synaptophysin, TNF-α, IL-1β, and GFAP antibodies. AD-specific markers, amyloid-β (1–42), and p-Tau were examined within PsEVs using Western blot and ELISA.

**Results:**

Our findings reveal higher concentrations of PsEVs in AD and MCI compared to AMC (*p* < 0.0001). Amyloid-β (1–42) expression within PsEVs is significantly elevated in MCI and AD compared to AMC. We could also differentiate between the amyloid-β (1–42) expression in AD and MCI. Similarly, PsEVs-derived p-Tau exhibited elevated expression in MCI compared with AMC, which is further increased in AD. Synaptophysin exhibited downregulated expression in PsEVs from MCI to AD (*p* = 0.047) compared to AMC, whereas IL-1β, TNF-α, and GFAP showed increased expression in MCI and AD compared to AMC. The correlation between the neuropsychological tests and PsEVs-derived proteins (which included markers for synaptic integrity, neuroinflammation, and disease pathology) was also performed in our study. The increased number of PsEVs correlates with disease pathological markers, synaptic dysfunction, and neuroinflammation.

**Conclusions:**

Elevated PsEVs, upregulated amyloid-β (1–42), and p-Tau expression show high diagnostic accuracy in AD. The downregulated synaptophysin expression and upregulated neuroinflammatory markers in AD and MCI patients suggest potential synaptic degeneration and neuroinflammation. These findings support the potential of PsEV-associated biomarkers for AD diagnosis and highlight synaptic dysfunction and neuroinflammation in disease progression.

**Supplementary Information:**

The online version contains supplementary material available at 10.1186/s12916-024-03475-z.

## Background

The progressive neurodegenerative condition known as Alzheimer’s disease (AD) is characterized by cognitive decline as a result of the formation of amyloid-β (Aβ) plaques, neurofibrillary tangles (NFTs), and chronic neuroinflammation that leads to neurodegeneration [1–3]. Synapse loss is a crucial pathophysiological event in disease progression, and synaptic proteins have been extensively studied due to earlier perturbations [4, 5]. The pathological hallmark of AD, amyloid-β plaques, originates from the imprecise cleavage of the amyloid precursor protein (APP) by β-secretase (BACE1) and γ-secretase generating amyloid-β peptide forms [6–9]. Primary amyloid-β peptide forms are Aβ40 and Aβ42, where the majority of the amyloid-β plaques in AD brains are composed of Aβ42 [10]. Many point mutations in APP and γ-secretase cause familial early-onset AD, favoring Aβ42 formation, causing amyloid-β peptides prone to aggregate as fibrils and plaques [9, 11–14]. Hyperphosphorylation of tau causes the formation of NFTs. The combined effect of accumulation of NFTs, amyloid-β fibrils, and plaques leads to neuronal function loss and cell death [15, 16]. Aβ plaques activate immune receptors on microglia, thereby releasing pro-inflammatory cytokines and chemokines that mediate neuroinflammation, which, if it reaches a chronic level, causes damage to brain cells, including axonal demyelination and synaptic pruning [17–23]. In addition to these, other proteins, including the neurofilament light (NFL) protein, glial fibrillary acidic protein (GFAP), and synaptic proteins, have also been identified as AD biomarkers [24–28]. Understanding the intricate dynamics of AD in terms of its varied pathophysiological manifestations, such as neuroinflammation, synaptic loss, and proteinopathy, is essential for developing potential therapeutic interventions for AD and biomarker discovery. In clinical practice, cognitive assessment tools such as the Addenbrooke’s Cognitive Examination (ACE-III) and Mini-Mental State Examination (MMSE) are used to diagnose AD. These tools evaluate verbal fluency and temporal orientation, although results may be influenced by subject bias [29–31].

In recent years, small extracellular vesicles (sEVs) or exosomes have been acknowledged as crucial mediators of communication and signaling within the body, contributing significantly to the transmission of cellular cargo in various health and disease states. They also play a notable role in disseminating protein aggregates associated with neurodegenerative diseases [32]. sEVs are bi-layered membrane vesicles that have a heterogeneous group of (< 200 nm in diameter) that are found in different human body fluids, including blood, urine, saliva, and ascites, and that are actively released by all cell types [33–35]. For their functions in various physiological and pathological circumstances, sEVs are the most extensively researched type of EV [36–38]. sEVs exchange information between cells by transferring bioactive components (nucleic acids and proteins) [39]. As the sEVs’ composition bears the molecular signature of the secreting cell and bears an intrinsic property of transversing the blood–brain barrier (BBB) in both directions [40, 41], they are a target of constant research in neurodegenerative disease. Furthermore, sEVs released by neuronal cells are crucial in transmitting signals to other nerve cells, influencing central nervous system (CNS) development, synaptic activity regulation, and nerve injury regeneration. Moreover, sEVs exhibit a dual function in neurodegenerative processes, as sEVs not only play an essential role in clearing misfolded proteins, thereby exerting detoxifying effects and providing neuroprotection [42]. On the other hand, they also have the potential to participate in the propagation and aggregation of misfolded proteins, particularly implicated in the pathological spread of Tau aggregates as indicated by both in vitro and in vivo studies [43]. As a protective mechanism, astrocytes (most abundant glial cells) accumulate at the locations where Aβ peptides are deposited, internalizing and breaking down aggregated peptides [44]. However, severe endosomal–lysosomal abnormalities arise in astrocytes when a significantly large amount of Aβ accumulates within astrocytes for a prolonged period without degradation [45, 46]. Astrocytes then release engulfed amyloid-β (1-42) protofibrils through exosomes, leading to severe neurotoxicity to neighboring neurons [44]. Additionally, it has been found that the release of amyloid-β by microglia in association with large extracellular vesicles (Aβ-lEVs) damages synaptic plasticity and modifies the architecture of the dendritic spine [47]. Thus, sEVs can be a compelling subject for the investigation to understand AD’s inflammation and synaptic dysfunction [48–52].

In this study, we reported that protein levels are associated with AD pathology, neuroinflammation, and synaptic dysfunction in plasma-derived small extracellular vesicles (PsEVs). Our objective was to understand the pathophysiological process, neuroinflammation, synaptic dysfunction, and Aβ pathology through sEVs. Our study revealed a significant correlation between the concentration of cargo proteins derived from PsEVs and clinical diagnosis concerning ACE-III and MMSE scores. Furthermore, the levels of these studied proteins within PsEVs could differentiate between patients with MCI and AD. Thus, our study sheds light on the potential of PsEVs in understanding AD dynamics and offers insights into the underlying mechanisms of disease progression.

## Methods

### Subject recruitment

A total of *n* = 35 AD patients and *n* = 25 subjects with MCI were recruited from the Memory Clinic, Department of Geriatrics, All India Institute of Medical Sciences, New Delhi, India. Additionally, *n* = 30 healthy AMC (volunteers) were recruited. The inclusion criteria were as follows: a clinical diagnosis of MCI and AD patients using ACE-III and MMSE tests. The exclusion criteria encompass medical conditions such as cancer, autoimmune disorders, liver disease, hematological disorders, or stroke, as well as psychiatric conditions, substance abuse, or any impediment to participation. Controls were healthy, age-matched adults without neurological symptoms. AMC was 60–71, MCI was 65–79, and AD was 70–80 years of age range (Table 1). Neuropsychological scores, viz., ACE-III and MMSE, were recorded before subject selection.

**Table 1:** Demographic details of samples

	**Age-matched control (AMC)**	**Mild cognitive impairment (MCI)**	**Alzheimer’s disease (AD)**
Age (mean ± SEM)	67.53 ± 1.154	73.08 ± 1.501	74.09 ± 0.8100
Male percentage	68.7%	60.00%	62.85%
ACE-III (mean ± SEM)	–––	69.04 ± 2.323	39 ± 3.511
MMSE (mean ± SEM)	–––	24.52 ± 0.726	12.86 ± 1.146
Aβ1-42 (mean ± SEM)	7.889 ± 0.5326	23.87 ± 1.550	42.40 ± 1.256
p-Tau (mean ± SEM)	21.99 ± 0.5978	29.09 ± 1.248	42.20 ± 0.7646
TNF-α (mean ± SEM)	66.86 ± 11.06	167.9 ± 10.04	359.7 ± 9.465
IL-1β (mean ± SEM)	10.13 ± 1.166	27.09 ± 1.691	62.27 ± 1.37
GFAP (mean ± SEM)	19.06 ± 1.727	28.34 ± 0.994	39.27 ± 2.886
Synaptophysin (mean ± SEM)	49.06 ± 1.70	36.85 ± 2.614	21.51 ± 1.943

Addenbrooke Cognitive Examination (ACE-III) and Mini Mental State Examination (MMSE), amyloid-β (1–42), phospho-Tau (p-Tau), tumor necrosis factor-alpha (TNF-α), interleukin 1 beta (IL-1β), glial fibrillary acidic protein (GFAP), and synaptophysin values are represented as mean ± SEM

### Study ethical approval

The institutional ethics committee of All India Institute of Medical Sciences, New Delhi, India, granted the study ethical permission. The study has been granted the ethical approval number IECPG-670/25.08.2022. Following the acquisition of the written informed consent, all participants were enrolled.

### Sample collection

One milliliter of blood was drawn from each participant using venipuncture, and blood collection vials were kept on ice during collection. The blood was centrifuged at 1700 g for 20 min at 4 °C to remove the cells, and the straw-colored plasma was collected. It was further clarified by centrifuging for 30 mi at 4 °C at 10,000 g. Finally, cleared plasma was stored at − 80 °C until further use. The samples were used for the downstream experiment after being thawed on ice and centrifuged at 10,000 g.

### Isolation of PsEVs

The PsEVs were extracted by chemical-based precipitation from the plasma samples of AD patients, MCI patients, and AMC, as discussed previously [53, 54]. In brief, 180 μL of plasma sample was used and filtered with 0.22 μm filter (SFNY25R, Axiva), followed by overnight incubation with the chemical precipitant (14% polyethylene glycol 6000) (807,491, Sigma). The samples underwent an hour-long, 13,000 g centrifugation at 4 °C the next day. Before being resuspended in 200 μL of 1X PBS (ML116-500ML, HiMedia), the pellet was first cleaned twice with 1X PBS. Before downstream experiments, the sEVs-enriched fraction was further filtered through a 100-kDa filter (UFC5100, Millipore).

### Nanoparticle tracking analysis (NTA)

5000-fold dilution in 1X-PBS buffer was used for the NTA of PsEVs. In the ZetaView Twin system (Particle Metrix, Germany) sample chamber, 1 mL of diluted PsEVs sample was introduced. The following parameters were used throughout three cycles of scanning 11 cell locations each, and 60 frames per position were collected (video setting: high, focus: autofocus, shutter: 150, 488 nm internal laser, camera sensitivity: 80, cell temperature: 25 °C. CMOS cameras were used for recording, and the built-in ZetaView Software 8.05.12 (Particle Metrix, Germany) was used to analyze: 10 nm as minimum particle size, 1000 nm as maximum particle size, and 30 minimum particle brightness.

### Transmission *electron* microscopy for morphological characterization

Transmission electron microscopy was employed to investigate PsEVs’ ultrastructural morphology. The resultant PsEVs pellet was diluted with PBS using 0.1 M phosphate buffer (pH 7.4). A carbon-coated copper grid of 300 mesh (01843, Ted Pella) was used to adsorb the separated PsEVs at room temperature for 30 min. After blot-drying, the adsorbed grids were dyed. For 10 s, 2% aqueous uranyl acetate solution (81,405, SRL Chem) as negative staining. After blotting the grids, they were inspected using a Talos S transmission electron microscope (ThermoScientific, USA).

### Western blot

Based on the initial volume of biofluid input, all samples were normalized, i.e., 180 μL and the sample loading dye (2 × Laemmle Sample buffer) was mixed with PsEVs sample, and 20 μL equal volume was loaded to run on an 8–12% SDS PAGE [53, 55]. After the completion of SDS-PAGE, protein from the gel was subjected to the Wet transfer onto the PVDF membrane of 0.22 μm (1,620,177, BioRad). The membrane-blocking with 3% bovine serum albumin (BSA) (D0024, BioBasic) in Tris (TB0194, BioBasic) base saline containing 0.1% of Tween 20 (65,296, SRL Chem) (TBST) using the BioRad Western blotting apparatus (BioRad, USA). Following this, overnight incubation of primary antibodies of CD63 (10628D, Invitrogen), CD81 (PA5-86,534, Invitrogen), TSG101 (MA1-23,296, Invitrogen), L1CAM (MA1-46,045, Invitrogen), synaptophysin (ADI-VAM-SV011-D, Enzo life sciences), GFAP (A19058, Abclonal), amyloid-β (1–42) oligomer (AHB0052, Invitrogen), phospho-Tau (s396) (35–5300, Invitrogen), interleukin 1β (IL-1β) (PA5-95,455, Invitrogen), tumor necrosis factor α (TNF-α) (E-AB-33121, Elabscience), and β-actin (AM4302, Invitrogen) were done at 4 °C. The membranes were washed with TBST buffer four times before at RT incubating with HRP-conjugated secondary antibodies, anti-rabbit (AB6721, Abcam), anti-mouse (31,430, Invitrogen). The Femto LUCENT™ PLUS-HRP kit (AD0023, GBiosciences) was used to develop the blot for visualizing the protein bands utilizing the method of enhanced chemiluminescence.

### Enzyme-linked Immunosorbent Assay (ELISA)

According to the previous protocol, ELISA was carried out. [53]. PsEV samples were subjected to freeze–thaw cycles; next, PsEVs were ultrasonicated for two minutes, with a 30-s on-and-off cycle, at an amplitude of 25. Following this, they underwent a 10-min centrifugation at 10,000 g, at 4 °C, and the obtained supernatant was used. The samples were kept at 37 °C before loading into the ELISA plates. The bicinchoninic acid (BCA) protein assay kit (23,225, ThermoFisher Scientific) was used to quantify the total protein concentration using BSA (D0024, BioBasic) as a reference. The ELISA kit was used to detect the presence of protein in 100 μL of PsEV sample are as follows: amyloid-β (1–42) (E-EL-H0543, ELabsciences), p-Tau (s-396) (E-EL-H5314, ELabsciences), IL-1β (ITLK01270, GBiosciences), TNF-α (ITLK01190, GBiosciences), GFAP (E-EL-H6093, ELabsciences), and synaptophysin (E-EL-H2014, ELabsciences). The manufacturer’s instructions were followed for every step of the process. A 96-well microplate spectrophotometer (SpectraMax i3x Multi-Mode Microplate Reader, Molecular devices) was used to measure the absorbance at 450 nm.

### Data and statistical analysis

The mean age values, ACE-III score, and MMSE score were ascertained using descriptive statistical analysis Table 1. GraphPad Prism 8.0 was used for statistical data analysis, including NTA concentration, Western blotting densitometric analysis, and ELISA. Unpaired student *t*-test and ANOVA were used for group analysis, and statistical significance was determined. *p* < 0.05 was used to assess significance. The Image J software (NIH, USA) was used for the densitometry analysis. The receiver operating characteristic (ROC) curve was used to analyze the efficiency of distinguishing the case from controls. Correlation analysis was conducted between the concentration of PsEVs and the levels of ELISA proteins, including amyloid-β (1–42), p-Tau, IL-1β, TNF-α, GFAP, and synaptophysin, and additionally between the PsEVs-derived levels of amyloid-β (1–42) β1-42, p-Tau, IL-1β, TNF-α, GFAP, and synaptophysin with ACE-III and MMSE values. ROC curve is a probability curve utilized to assess the accuracy of a test. The test’s ability to distinguish between groups is indicated by the area under the curve (AUC), which acts as a quantitative measure of separability. An outstanding test typically exhibits an AUC close to 1, signifying a high level of separability. Conversely, a subpar test tends to have an AUC closer to 0, indicating a poor ability to distinguish between the two classes.

## Results

### Characterization and validation of isolated sEVs

**Fig. 1:**
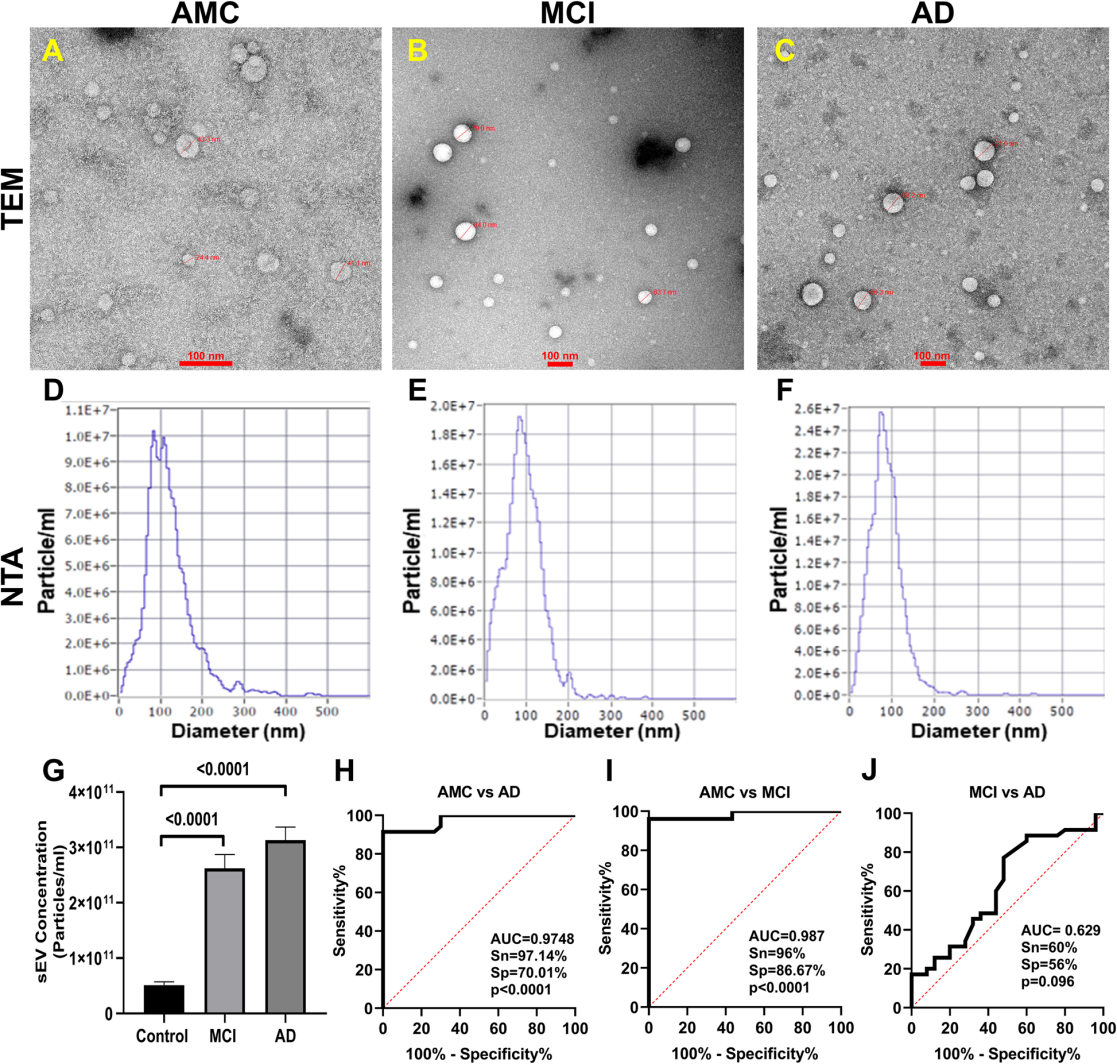
Isolation and analysis of PsEVs. The isolated PsEV morphology characterize by transmission electron microscopy from age-matched healthy controls (AMC) (**A**), mild-cognitive impairment (MCI) patients (**B**), and Alzheimer’s disease (AD) (**C**). The size distribution of PsEVs subpopulation (nm) versus the concentration (particle/ml) in AMC (**D**), individuals with MCI (**E**), and AD (**F**). Comparison of the sEVs concentration of AD, MCI, and AMC patients (**G**). Receiver operating characteristic (ROC) curve of PsEVs concentration in AMC v/s AD (**H**), AMC v/s MCI (**I**), and MCI v/s AD (**J**) (scale bar 100 nm)

**Fig. 2:**
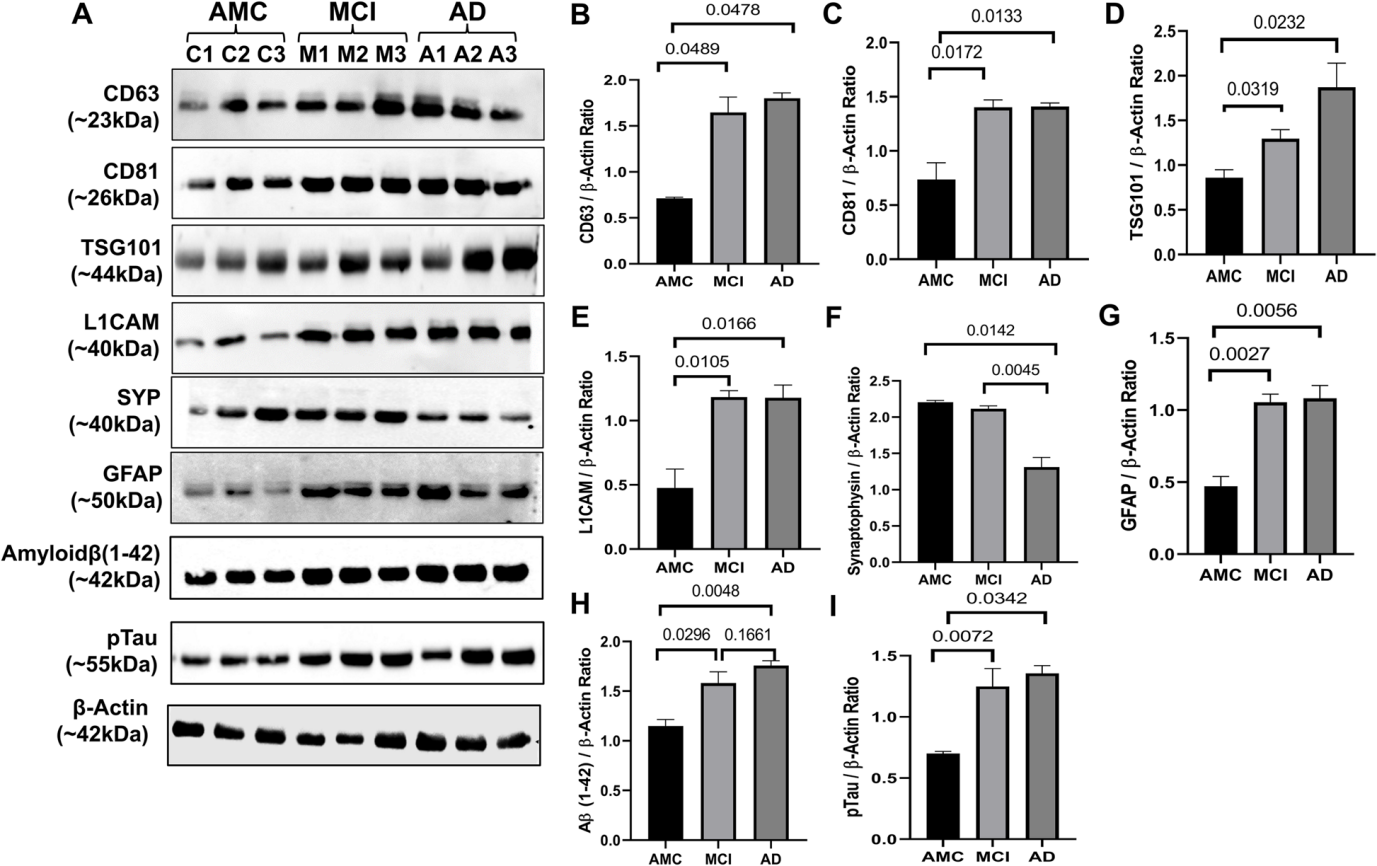
Validation of PsEVs expression analysis of different markers in PsEVs in age-matched controls (AMC), mild cognitive impairment (MCI), and Alzheimer’s disease patients (AD) (**A**). Densitometric analysis of CD63 (**B**), densitometric analysis of CD81 (**C**), densitometric analysis of TSG101 (**D**), densitometric analysis of L1CAM (**E**), densitometric analysis of synaptophysin (**F**), densitometric analysis of GFAP (**G**), and densitometric analysis of amyloid-β (1–42) oligomer (**H**). All densitometric values were normalized against β-actin

PsEVs were isolated, characterized, and validated following Minimal Information for Studies of Extracellular Vesicles (MISEV) 2018 guidelines, which suggest a protocol for documenting work specifically with extracellular vesicles [56]. PsEVs from AMC, MCI, and AD subjects were morphologically characterized by transmission electron microscopy, and spherical lipid bi-layered vesicles were observed in the size range of sEVs (Fig. 1A–C). In Fig. 1D–F, the size distribution and concentration of PsEVs were observed in the size range of 30–200 nm in diameter by NTA, which is within the sEVs’ size range. The mean concentration of PsEVs in AMC, MCI, and AD patients were 5.12E + 10, 2.6E + 11, and 3.13E + 11 particle/ml, respectively, with higher concentrations of PsEVs in MCI and AD than in AMC (*p* < 0.0001) (Fig. 1G). To differentiate AD from AMC, ROC and AUC analyses were performed where the AUC = 0.9748, with a sensitivity of 97.14% and specificity of 70.01% (Fig. 1H), while in AMC versus MCI, AUC = 0.987, sensitivity of 96% and specificity of 86.67% (Fig. 1I). Furthermore, we could also differentiate between MCI and AD, AUC = 0.629, sensitivity of 60%, and specificity of 56% (Fig. 1J). Validation of PsEVs was done using immunoblot for sEVs-specific markers (CD63, CD81, and TSG101), which showed a significant increase in expressions in MCI and AD than in AMC (CD63, *p* = 0.0489, 0.0478 (Additional File 1: Fig. S1); CD81, *p* = 0.0172, 0.0133 (Additional File 1: Fig. S2); TSG101 *p* = 0.0240, 0.0329 (Additional File 1: Fig. S3)) for AD and MCI respectively (Fig. 2A–D). Additionally, higher L1CAM (neuron-associated marker) expression was observed in MCI (*p* = 0.0100) and AD (*p* = 0.0184) (Additional File 1: Fig. S4) compared to AMC (Fig. 2E). All densitometric values were normalized against β-actin, which was used as a loading control (Additional File 1: Fig. S7).

### Differential expression of amyloid-β (1–42), p-Tau, synaptophysin, GFAP markers, and levels of IL-1β and TNF-α in PsEVs

**Fig. 3:**
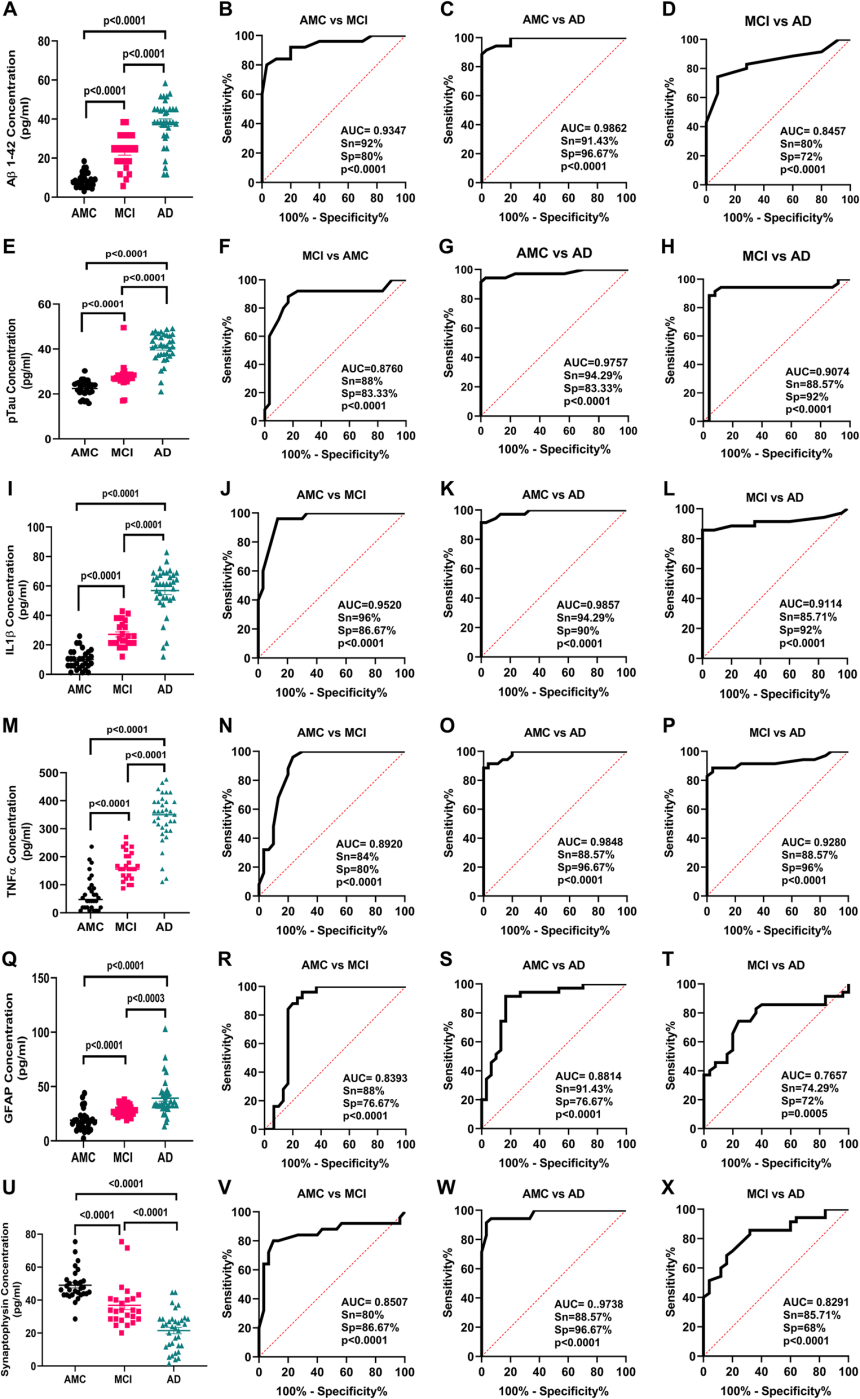
PsEVs derived amyloid-β (1–42), p-Tau, IL-1β, TNF-α, GFAP, and synaptophysin protein concentration was measured. ELISA results in **A** shows levels of PsEVs amyloid-β (1–42) in AMC, MCI, and AD and receiver operating characteristic (ROC) curve of PsEVs concentration in AMC v/s MCI (**B**), AMC v/s AD (**C**), and MCI v/s AD (**D**). Similarly, p-Tau concentration in AMC, MCI, and AD (**E**), ROC curve of PsEVs concentration in AMC v/s MCI (**F**), AMC v/s AD (**G**), and MCI v/s AD (**H**). PsEVs derived IL-1β concentration in AMC, MCI and AD (**I**), ROC curve of PsEVs concentration in AMC v/s MCI (**J**), AMC v/s AD (**K**), and MCI v/s AD (**L**). PsEVs derived TNF-α concentration in AMC, MCI and AD (**M**), ROC curve of PsEVs concentration in AMC v/s MCI (**N**), AMC v/s AD (**O**), and MCI v/s AD (**P**). Similarly, GFAP concentration in AMC, MCI, and AD (**Q**), ROC curve of PsEVs concentration in AMC v/s MCI (**R**), AMC v/s AD (**S**), and MCI v/s AD (**T**). For PsEVs-derived synaptophysin concentration in AMC, MCI, and AD (**U**), ROC curve of PsEVs concentration in AMC v/s MCI (**V**), AMC v/s AD (**W**), and MCI v/s AD (**X**). Abbreviations: AMC, age-matched control; MCI, mild-cognitive impairment patients; AD, Alzheimer’s disease patients; TNF-α, tumor necrosis factor-alpha; GFAP, glial fibrillary acidic protein

Using ELISA, we measured levels of amyloid-β (1–42) and p-Tau in PsEVs from AMC, MCI, and AD patients. The significant increase of amyloid-β (1–42) and p-Tau among the groups (Fig. 3A–H). Amyloid-β (1–42) levels were higher in MCI compared to AMC (*p* < 0.0001) and more significant in AD than in MCI and AMC (*p* < 0.0001) (Fig. 3A). Similarly, in comparison to MCI and AMC, p-Tau levels were significantly higher in AD (*p* < 0.0001) (Fig. 3E). Similar levels of both markers were found in their Western blots (Fig. 2). We checked GFAP (astrocytic marker) and proinflammatory cytokines (TNF-α and IL-1β) to evaluate neuroinflammation. For proinflammatory markers, IL-1β and TNF-α levels showed a significant increase among the three groups (*p* < 0.0001 for IL-1β and TNF-α) (Fig. 3I, M). When comparing AD to MCI and AMC, the GFAP concentration in PsEVs was significantly higher (*p* < 0.0001) (Fig. 3Q). Similar trends were observed with Western blot analysis (Fig. 2, Additional File 1: Fig. S6, S9). Their elevated levels suggest prominent neuroinflammatory conditions contributing to potential neuronal damage. The elevated levels of these neuroinflammatory markers could be due to the activation of astrocytes and microglia and the subsequent increase in the secretion of PsEVs containing proinflammatory proteins, which suggests prominent neuroinflammatory conditions that may contribute to neuronal damage [57]. While synaptophysin concentration in PsEVs was downregulated in AD and MCI compared to AMC (*p* < 0.0001) in ELISA (Fig. 3U), it shows synaptic dysfunction. We also checked synaptophysin levels in PsEVs in Western blotting, finding it was downregulated in AD compared to MCI and AMC (*p* = 0.0045, 0.0142), indicating synaptic degeneration in AD (Fig. 2, Additional File 1: Fig. S5). In MCI, synaptophysin levels did not significantly differ from AMC (Fig. 2F). This aligns with synaptic loss in AD, reflected in lower neuropsychological test scores indicating more pronounced cognitive impairment compared to MCI and AMC.

### Determining the diagnostic potential of PsEVs-derived amyloid-β (1–42), p-Tau, IL-1β, TNF-α, GFAP and synaptophysin

We observed the levels of amyloid-β (1–42) and p-Tau in PsEVs, where the increase in amyloid-β (1–42) and p-Tau levels underscores their potential as biomarkers of MCI and AD. The diagnostic efficacy of amyloid-β (1–42) by ROC analysis was observed for AMC vs MCI [AUC = 0.9347, *p* < 0.0001, sensitivity (Sn) = 92%, specificity (Sp) = 80%] (Fig. 3B), AMC vs AD (AUC = 0.9862, *p* < 0.0001, Sn = 91.43%, Sp = 96.67%) (Fig. 3C), and MCI vs AD (AUC of 0.8457, *p* < 0.0001, Sn = 80%, and Sp = 72%) (Fig. 3D). Similarly, diagnostic efficacy of p-Tau by ROC analysis was observed for AMC vs MCI (AUC = 0.8760, *p* < 0.0001, Sn = 88%, Sp = 83.33%) (Fig. 3F), AMC vs AD (AUC = 0.9757, *p* < 0.0001, Sn = 94.29%, Sp = 83.33%) (Fig. 3G), and MCI vs AD (AUC of 0.9074, *p* < 0.0001, Sn = 88.57%, and Sp = 92%) (Fig. 3H). So, we observed that the pathological hallmarks of the disease, viz., amyloid-β (1–42) and p-Tau levels, are increased significantly in PsEVs cargo of AD and MCI groups.

Furthermore, we also checked GFAP, TNF-α, IL-1β, and synaptophysin in PsEVs from MCI and AD groups. The diagnostic efficacy of IL-1β by ROC analysis was observed for AMC vs MCI (AUC = 0.9520, *p* < 0.0001, Sn = 96%, Sp = 86.67%) (Fig. 3J), AMC vs AD (AUC = 0.9857, *p* < 0.0001, Sn = 94.29%, Sp = 90%) compared to AMC (Fig. 3K), MCI vs AD (AUC = 0.9114, *p* < 0.0001, Sn = 85.71%, Sp = 92%) (Fig. 3L). Similarly, diagnostic efficacy of TNF-α by ROC analysis was observed for AMC vs MCI (AUC = 0.8920, *p* < 0.0001, Sn = 84%, Sp = 80%) (Fig. 3N), AMC vs AD (AUC = 0.9848, *p* < 0.0001, Sn = 88.57%, Sp = 96.67%), and MCI vs AD (AUC = 0.9280, *p* < 0.0001, Sn = 88.57%, Sp = 96%) (Fig. 3P). So, we observed an elevated expression of neuroinflammatory markers within the PsEVs isolated from the AD and MCI groups.

GFAP is an activation marker of astroglia, and in AD, this activation is associated with synaptic dysfunction [58]. In PsEVs, the diagnostic efficacy of GFAP by ROC analysis was observed as for AMC vs MCI (AUC = 0.8393, *p* < 0.0001, Sn = 88%, Sp = 76.67%) (Fig. 3R), AMC vs. AD (AUC = 0.8814, *p* < 0.0001, Sn = 91.43%, Sp = 76.67%) compared to AMC (Fig. 3S); MCI vs AD (AUC = 0.7657, *p* < 0.0001, Sn = 74.29%, Sp = 72%) (Fig. 3T). In addition to this, we also checked the level of presynaptic protein, i.e., synaptophysin, within the PsEVs, as the level of synaptophysin correlates with cognitive decline in AD [59]. The diagnostic efficacy of synaptophysin by ROC analysis was observed as follows for AMC vs MCI (AUC = 0.8507, *p* < 0.0001, Sn = 80%, Sp = 86.67%) (Fig. 3V), AMC vs AD (AUC = 0.9738, *p* < 0.0001, Sn = 88.57%, Sp = 96.67%) compared to AMC (Fig. 3W); MCI vs AD (AUC = 0.8291, *p* < 0.0001, Sn = 85.71%, and Sp = 68%) (Fig. 3X). Table 2 summarizes all the AUC, sensitivity, specificity, and *p*-value values for all the PsEVs-derived proteins.

**Table 2:** Determining the diagnostic potential of PsEVs-derived amyloid-β (1–42), p-Tau, IL-1β, TNF-α, GFAP, and synaptophysin

S. no	Markers	Subject groups	AUC values	Sensitivity (%)	Specificity (%)	*p*-value
1	Amyloid-β (1–42)	AMC v/s MCI	0.9347	92	80	< 0.0001
2		AMC v/s AD	0.9862	91.43	96.67	< 0.0001
3		AD v/s MCI	0.8457	80	72	< 0.0001
4	p-Tau	AMC v/s MCI	0.8760	88	83.33	< 0.0001
5		AMC v/s AD	0.9757	94.29	83.33	< 0.0001
6		AD v/s MCI	0.9074	88.57	92	< 0.0001
7	IL-1β	AMC v/s MCI	0.9520	96	86.67	< 0.0001
8		AMC v/s AD	0.9857	94.29	90	< 0.0001
9		AD v/s MCI	0.9114	85.71	92	< 0.0001
10	TNF-α	AMC v/s MCI	0.8920	84	80	< 0.0001
11		AMC v/s AD	0.9848	88.57	96.67	< 0.0001
12		AD v/s MCI	0.9280	88.57	96	< 0.0001
13	GFAP	AMC v/s MCI	0.8393	88	76.67	< 0.0001
14		AMC v/s AD	0.8814	91.43	76.67	< 0.0001
15		AD v/s MCI	0.7657	74.29	72	0.0005
16	Synaptophysin	AMC v/s MCI	0.8507	80	86.67	< 0.0001
17		AMC v/s AD	0.9738	88.57	96.67	< 0.0001
18		AD v/s MCI	0.8291	85.71	68	< 0.0001

*Abbreviations*: *TNF-α* Tumor necrosis factor-alpha, *GFAP* Glial fibrillary acidic protein. Receiver operating characteristic (ROC) curves of all PsEVs derived-protein were performed, where the area under the ROC curve (AUC) is tabulated with *p*-value

### Correlations of PsEVs concentration values with protein levels of amyloid-β (1–42), p-Tau, IL-1β, TNF-α, GFAP, and synaptophysin in PsEVs

**Fig. 4:**
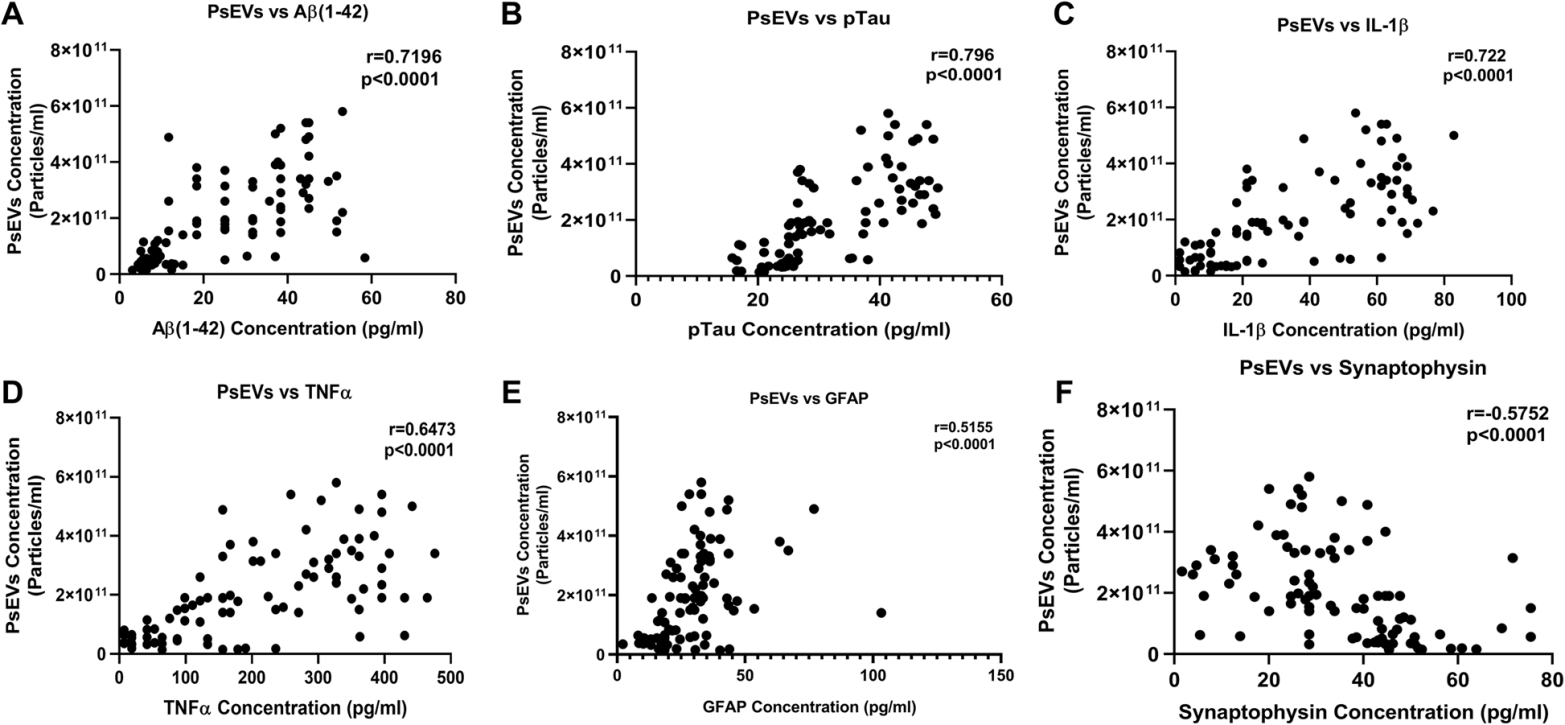
Correlation analysis between PsEVs concentration and PsEVs derived AD pathology markers. The correlation between PsEVs concentration with the amyloid-β (1–42) (**A**), p-Tau (**B**), IL-1β (**C**), TNF-α (**D**), GFAP (**E**), and synaptophysin (**F**). Abbreviations: p-Tau, Phospho-Tau, TNF-α, tumor necrosis factor-alpha; GFAP, glial fibrillary acidic protein. Spearman correlation was used for correlation analysis

As we found an elevated number of PsEVs in the diseased condition, we performed a correlation analysis between PsEVs concentration and the amyloid-β (1–42) level, p-Tau, IL-1β, and TNF-α within PsEV. We found that PsEV concentration was positively correlated with all the protein levels except synaptophysin, which showed a negative correlation (Fig. 4). In these correlations, amyloid-β (1–42) was positively correlated (*r* = 0.7196, *p* < 0.0001) (Fig. 4A); p-Tau positively correlates (*r* = 0.7960, *p* < 0.0001) (Fig. 4B); IL-1β also showed positive correlation (*r* = 0.7220, *p* < 0.0001) (Fig. 4C); and TNF-α also showed positive correlation (*r* = 0.6473, *p* < 0.0001) (Fig. 4D). GFAP showed a weak correlation with PsEVs concentration (*r* = 0.5155, *p* < 0.0001) (Fig. 4E), and synaptophysin showed a weak correlation (*r* = 0.5752, *p* < 0.0001) (Fig. 4F).

### Correlations of ACE-III and MMSE scores with protein levels of amyloid-β (1–42), p-Tau, IL-1β, and TNF-α in PsEVs

**Fig. 5:**
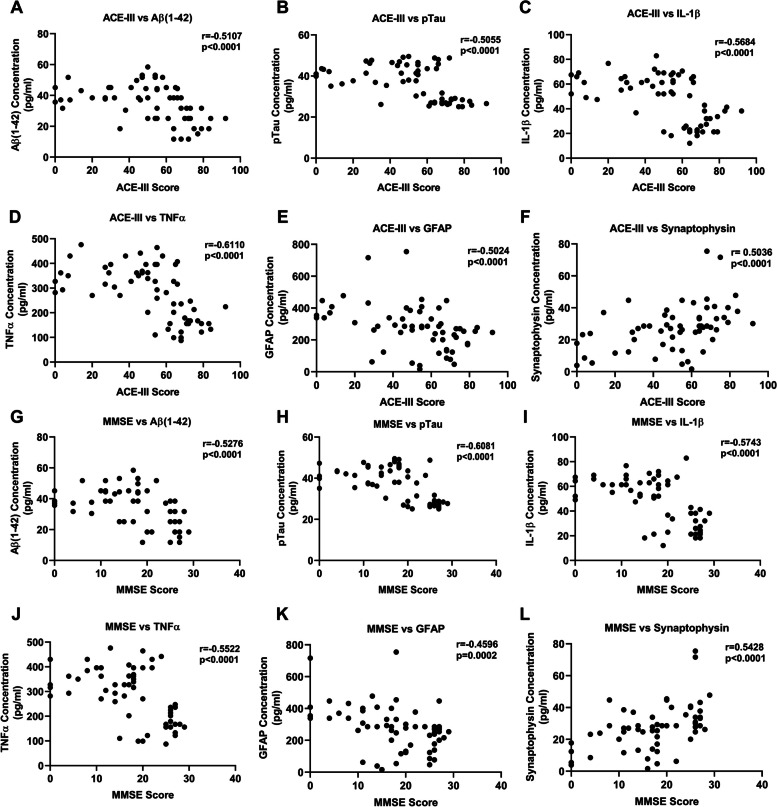
Correlation between neuropsychological test (ACE-III and MMSE) and PsEV-derived AD pathology markers. Amyloid-β (1–42) β, p-Tau, IL-1β, TNF-α, GFAP, and synaptophysin protein concentration. **A**–**F** Correlation between ACE-III scores and amyloid-β (1–42) (**A**), pTau (**B**), IL-1β (**C**), TNF-α (**D**), GFAP (**E**), and synaptophysin (**F**) protein concentration. **G**–**L** A correlation between MMSE Score and amyloid-β (1–42) (**G**), p-Tau (H), IL-1β (**I**), TNF-α (**J**), GFAP (**K**), and synaptophysin (**L**) protein concentration. Abbreviations: ACE-III, Addenbrooke Cognitive Examination; MMSE, Mini-Mental State Examination; p-Tau, Phospho-Tau; TNF-α, tumor necrosis factor-alpha; GFAP, glial fibrillary acidic protein. Spearman correlation was used for correlation analysis

We performed a correlation analysis between ACE-III and MMSE values with the level of amyloid-β (1–42), p-Tau, IL-1β, TNF-α, GFAP, and synaptophysin (Fig. 5). We found that ACE-III and MMSE values were negatively correlated with all the protein levels except synaptophysin, which showed a positive value for the correlation coefficient. ACE-III values showed a negative correlation with amyloid-β (1–42) (*r* =  − 0.5107, *p* < 0.0001) (Fig. 5A), p-Tau (*r* =  − 0.5055, *p* < 0.0001) (Fig. 5B), IL-1β (*r* =  − 0.5684, *p* < 0.0001) (Fig. 5C), and TNF-α (*r* =  − 0.6110, *p* < 0.0001) (Fig. 5D). ACE-III values showed a negative correlation with GFAP (*r* =  − 0.5024, *p* < 0.0001) (Fig. 5E), while synaptophysin showed a positive correlation (*r* = 0.5036, *p* < 0.0001) (Fig. 5F). In the case of MMSE, the values were as follows: for amyloid-β (1–42) (*r* =  − 0.5276, *p* < 0.0001) (Fig. 5G), p-Tau (*r* =  − 0.6081, *p* < 0.0001) (Fig. 5H), IL-1β (*r* =  − 0.5743, *p* < 0.0001) (Fig. 5I), TNF-α (*r* =  − 0.5522, *p* < 0.0001) (Fig. 5J), GFAP (*r* =  − 0.4596 *p* = 0.0002) (Fig. 5K), and synaptophysin (*r* = 0.5428, *p* < 0.0001) (Fig. 5L). Table 3 summarizes all the values of Correlation coefficients for all the PsEVs-derived proteins.

**Table 3:** Correlation analysis of PsEVs-derived amyloid-β (1–42), p-Tau, IL-1β, TNF-α, GFAP, and synaptophysin with neuropsychological scores

S. no	**Markers**	**PsEVs concentration**	**ACE-III scores**	**MMSE scores**
1	Amyloid-β (1–42)	*r* = 0.7196	*r* = − 0.5107	*r* = − 0.5276
2	p-Tau	*r* = 0.7960	*r* = − 0.5055	*r* = − 0.6081
3	IL-1β	*r* = 0.7220	*r* = − 0.5684	*r* = − 0.5743
4	TNF-α	*r* = 0.6473	*r* = − 0.6110	*r* = − 0.5522
5	GFAP	*r* = 0.5155	*r* = − 0.5024	*r* = − 0.4596
6	Synaptophysin	*r* = 0.5356	*r* = 0.5036	*r* = 0.5428

viz., Addenbrooke Cognitive Examination (ACE-III) and Mini-Mental State Examination (MMSE) scores

*Abbreviations*: *TNF-α* Tumor necrosis factor-alpha, *GFAP* Glial fibrillary acidic protein. Spearman correlation was used for correlation analysis

## Discussion

In this study, we aimed to investigate the capacity of PsEVs to mirror pathological processes linked to AD and MCI. sEVs are extensively documented in the propagation of pathological processes associated with neurodegenerative and metabolic disorders [60]. The increased secretion of sEVs, coupled with the transmission of disease-related pathologies through sEVs-associated cargo, makes sEVs a viable candidate for understanding the physiological state of their originating cells, which is reflected in sEVs cargo [61]. To isolate the PsEVs, we employed a combined approach involving chemical precipitation followed by ultrafiltration, which effectively eliminates co-precipitants and minute protein contaminants such as albumin and LDL. We employed the neuronal protein L1CAM as a marker to ascertain the neuronal origin, although there is a debate surrounding its specificity for neuronal origin [62]. Nevertheless, in our study, the L1CAM marker is used to check for protein markers and not to confirm L1CAM affinity-based isolation. A two-step filtration procedure was used to accompany the sEV isolation method in our study to ensure high purity. Spherical lipid bi-layered vesicles within the typical size range of small extracellular vesicles (30–150 nm) were observed across AD, MCI, and AMC subjects (Fig. 1A–C). NTA was employed to study the size distribution of sEVs in AD, MCI, and AMC. We observed that the isolated PsEVs come within the size range of < 200 nm, and there was a notable increase in the number of particles in diseased conditions compared to the control group. (Fig. 1D–G).

Validation using sEVs-specific markers (CD63, CD81, and TSG101) demonstrated a noteworthy upregulation in MCI and AD, indicating PsEVs numbers are increased in disease conditions (Fig. 2A–D). Levels of sEV-specific markers in AD and MCI are elevated because PsEV numbers are increased in the disease condition. As documented by various studies in MCI and AD, there is an increase in cross-talk between different pathophysiological processes, which leads to an increase in sEVs number and sEVs specific marker as a cellular response to heightened cellular stress aggravating neuronal damage and synaptic dysfunction [33, 63, 64]. Neuroinflammation, a characteristic feature of AD and MCI, may lead to the release of sEVs with inflammatory markers. Synaptic dysfunction, evidenced by synaptic degeneration, could contribute to the increased sEV-specific markers, reflecting vesicle release in response to altered synaptic activity [9, 65]. Additionally, cells undergoing stress might activate compensatory mechanisms, and the elevated sEV-specific markers could signify communication for potential repair or damage mitigation. Therefore, the increase in sEV-specific markers may be linked to the progression of neurodegenerative processes, indicating ongoing pathological changes in the brain as the disease progresses. Additionally, the elevated expression of L1CAM, a neuron-associated marker, in MCI and AD further strengthens the association between PsEVs and neurodegenerative processes (Fig. 2E). Furthermore, our observations extend beyond AD and MCI, showing increased concentrations of sEVs in other health conditions where higher levels of these vesicles correlate with elevated levels of disease markers [53–55]. The results of our research provide valuable insight into the characterization, validation, and functional implications of plasma-derived small extracellular vesicles (PsEVs) in the context of AD and MCI. Our comprehensive analysis encompassed morphological, biochemical, and functional aspects, shedding light on the potential role of PsEVs as biomarkers and contributors to neurodegenerative processes.

For this purpose, we performed the ELISA of amyloid-β (1–42) in PsEVs, where we observed higher protein concentrations of amyloid-β (1–42) in MCI. At the same time, in AD, the concentration also significantly increased (Fig. 3A). In a similar study by A. Manolopoulos et al. [66], they studied levels of Aβ42, total Tau, and pro-brain-derived neurotrophic factor (BDNF) in both plasma neuron-derived extracellular vesicles (NDEVs) and plasma. The study reported a lack of correlation between the plasma and NDEVs, substantiating concerns about levels of the Aβ42 and total Tau measured in plasma originating from non-CNS sources. Multiple studies support the involvement of extracellular vesicles (EVs) in AD pathogenesis, where Aβ and Tau are released in association with EVs, influencing neuronal cell death and trans-synaptic spreading of the disease [7, 15, 54, 67]. A progressive elevation in PsEV levels of p-Tau was observed in MCI, reaching a significantly higher AD concentration (Fig. 3E). Previous research has revealed that p-tau alone effectively differentiates Frontotemporal Dementia (FTD) from AD with high specificity [68, 69]. In our study, the alone analysis of p-Tau and amyloid-β (1–42) proved effective in distinguishing patients with MCI from AMC (Table 2). Consequently, studies have reported that the elevation of p-Tau suggests the future likelihood of AD development [70]. This dual elevation in amyloid-β (1–42) and p-Tau levels highlights their potential utility as concurrent biomarkers associated with MCI and AD diagnosis, as indicated by our ROC analysis. Therefore, the investigation into PsEV content revealed significant alterations in key markers associated with AD pathology, viz., amyloid-β (1–42) and p-Tau, which are a well-established marker of AD and exhibit an elevated level in PsEVs from AD and MCI patients compared to AMC in our study.

Synaptic dysfunction is considered a core feature of AD. It is suggested to precede other pathophysiological events of AD rather than neurodegeneration, which manifests during the later stages of the disease [71]. Synaptic dysfunction interacts with other core pathophysiology events of AD, such as the amyloid-β cascade, tau pathology, and neuroinflammation, eventually progressing to irreversible neurodegeneration and atrophy [72, 73]. In this context, the synchronized exchange of proteins involved in these pathological processes between the CNS and neuronal-derived sEVs highlights the potential of sEVs as reliable carriers of pathophysiological cascade occurring at the pathological site [74]. In Fig. 3U, we observed downregulated synaptophysin levels, a synaptic vesicle marker, in AD PsEVs compared to MCI and AMC. This suggests synaptic degeneration, which has also been discussed in several studies [59, 63, 64]. Synaptic damage induced by amyloid-β deposition triggers a response from the glia to eliminate impaired synapses. As amyloid-β accumulates, the severity of synaptic dysfunction intensifies, leading to tau hyperphosphorylation and the formation of tau tangles. Our study’s findings contradict J. Utz et al. (2021), which showed increased synaptophysin levels in microvesicles isolated from cerebrospinal fluid (CSF) in AD [28]. This discrepancy could be due to different biofluid sources, cellular origins, or clearance mechanisms for synaptophysin in these compartments. Our study also differs from Utz J et al. (2021) as we have studied PsEVs compared to microvesicles; both differ in biogenesis, structure, and functions. Moreover, our study aligns with existing studies that reported lower synaptophysin levels in plasma neuronal-derived EVs. Goetzl et al. [75] investigated the synaptic protein levels in neuronal-derived exosomes in plasma (NDEs) of patients with FTD and AD, where the authors found significantly lower levels of synaptopodin, neurogranin, synaptophysin, and synaptotagmin-2 in both conditions compared to controls. Furthermore, our results also align with the overall synaptic loss seen in AD patient’s brains, where lower levels of synaptophysin in the hippocampus have been reported to correlate with cognitive decline in AD [59]. Our study found that no significant difference in synaptophysin levels between MCI and AMC was observed, indicating that synapse dysfunction is more pronounced due to neuronal loss in the advanced disease stage, and its indication is reflected in PsEVs. Since the PsEVs pool also contains neuronal-derived EVs, we interpolate that the reduction in synaptic proteins in brain tissue is reflected in our results.

IL-1β, a potent immunomodulating cytokine, has previously been identified as a trigger for various inflammatory mediators in astrocytes and neurons [76]. Consistent evidence from post-mortem AD brain studies indicates the prevalent overexpression of IL-1β, with immunohistochemical analyses revealing its localization to microglia around plaques [77]. Moreover, pro-inflammatory markers (IL-1β and TNF-α) were significantly higher in PsEVs from AD and MCI subjects, as evidenced by ELISA and Western blot findings in our study (Fig. 3I and M). Table 3 summarizes the correlation between PsEVs and neuroinflammatory markers. IL-1β plays a direct role in the pathophysiological changes associated with AD owing to its specific expression in the vicinity of plaques, and this localization suggests IL-1β as a mediator in the formation of plaques and tangles, thereby contributing to AD pathology [65]. TNF-α, another pro-inflammatory cytokine primarily secreted by activated macrophages and microglia, is recognized for its dual role in promoting cell survival and death in the central nervous system [78, 79].

The cytoskeletal GFAP is found in astrocytic cells [80]. Increased plasma GFAP levels could result from “reactive astrogliosis,” another term for aberrant astrocytic function brought on by damage to neurons [81]. According to research on animal and cell models, reactive astrocytes encircle and penetrate amyloid-β plaques, contributing to the amyloid-β pathological process [82, 83]. Research has demonstrated a correlation between amyloid-β burden, cognitive decline, and plasma GFAP [83]. PsEVs of GFAP were elevated in AD [27] and MCI (Fig. 3Q). It is well known that sEVs play a pivotal role in the progression of disease pathologies in neurodegenerative and metabolic diseases [33, 84]. The high levels of neuro-inflammatory markers (GFAP, TNF-α, and IL-1β) in PsEVs from MCI and AD subjects suggest a potential role of PsEVs in neuroinflammation. This activation of astrocytes and microglia precedes increased secretion of pro-inflammatory PsEVs and may contribute to neuronal damage and progressive cognitive impairment. Diseased conditions involve an increased secretion of sEVs and the cargo they carry, including pathological hallmark proteins or immunomodulatory cytokines [33].

Correlation analyses unveiled positive associations between PsEVs concentration and the protein levels of amyloid-β (1–42), p-Tau, IL-1β, TNF-α, GFAP, and synaptophysin (Fig. 4). Furthermore, our study also analyzed the correlation between cognitive examination scores (ACE-III and MMSE) and PsEV-associated protein levels (Fig. 5). The negative correlations observed imply that lower cognitive scores align with elevated levels of amyloid-β (1–42), p-Tau, IL-1β, and TNF-α in PsEVs Table 3. This implies a strong connection between PsEV biomarkers and cognitive decline, reinforcing that PsEVs could serve as valuable diagnostic and prognostic tools. These findings underscore the potential of PsEVs as reliable disease progression and pathology indicators. The robust correlations further support the hypothesis that PsEVs may actively participate in disseminating neurodegenerative signals.

Our study extensively studied the multiple pathophysiological processes associated with AD by checking the protein levels involved in these processes within PsEVs, including amyloid-β (1–42), p-Tau, neuroinflammatory markers (IL-1β, TNF-α, GFAP), and synaptic protein levels. This comprehensive approach enhances diagnostic accuracy by considering the synergistic effects of these processes, providing valuable insights into disease progression from MCI to AD. We have also performed a systematic comparison with MCI, which was lacking in previous studies. We observed a significant correlation between these investigated protein levels within PsEVs and neuropsychological tests, thus filling a research gap addressing the clinical relevance of these dysregulated pathophysiological processes. The observed downregulated synaptophysin levels in AD PsEVs compared to MCI and control subjects shed light on the combined role of neuroinflammation and proteinopathy in the cognitive decline observed as the disease progresses. This finding suggests that PsEVs may reflect synaptic degeneration, opening avenues for further exploration into the role of PsEVs in synaptic damage and dysfunction in neurodegenerative diseases.

## Conclusions

Our study provides a multifaceted examination of PsEVs, offering compelling evidence of their potential as biomarkers and functional contributors in AD. We have comprehensively discussed the synergistic role of synaptic dysfunction and neuroinflammation and their association with amyloid-β and tau pathologies within the PsEVs in AD progression. The pathophysiological conditions in the MCI and AD brain are reflected in PsEVs, as observed by the increased concentration of PsEVs containing disease-associated markers and markers for synaptic dysfunction and neuroinflammation. Therefore, the PsEVs can be exploited to understand the pathophysiological process involved in the progression and severity of MCI and AD.

### Supplementary Information

Additional file 1: Fig S1. [CD63 expression in age-matched controls (AMC), mild cognitive impairment (MCI), and Alzheimer’s disease (AD) and their densitometric analysis]. Fig S2. [CD81 expression in age-matched controls (AMC), mild cognitive impairment (MCI), and Alzheimer’s disease (AD) and their densitometric analysis]. Fig S3. [TSG101 expression in age-matched controls (AMC), mild cognitive impairment (MCI), and Alzheimer’s disease (AD) and their densitometric analysis]. Fig S4. [L1CAM expression in age-matched controls (AMC), mild cognitive impairment (MCI), and Alzheimer’s disease (AD) and their densitometric analysis]. Fig S5. [Synaptophysin (SYP) expression in age-matched controls (AMC), mild cognitive impairment (MCI), and Alzheimer’s disease (AD) and their densitometric analysis]. Fig S6. [Glial Fibrillary Acidic Protein (GFAP) expression in age-matched controls (AMC), mild cognitive impairment (MCI), and Alzheimer’s disease (AD) and their densitometric analysis]. Fig S7. [β-Actin expression in age-matched controls (AMC), mild cognitive impairment (MCI), and Alzheimer’s disease (AD) and their densitometric analysis]. Fig S8. [Amyloidβ-42 Oligomer expression in age-matched controls (AMC), mild cognitive impairment (MCI), and Alzheimer’s disease (AD) and their densitometric analysis]. Fig S9. [IL1β (A) and TNFα (B) expression in age-matched controls (AMC), mild cognitive impairment (MCI), and Alzheimer’s disease (AD) and their densitometric analysis]. Fig S10. [p-Tau expression in age-matched controls (AMC), mild cognitive impairment (MCI), and Alzheimer’s disease (AD) and their densitometric analysis].

## Data Availability

No datasets were generated or analysed during the current study.

## Abbreviations

ACE-III: Addenbrooke Cognitive Examination
AD: Alzheimer’s disease
AMC: Age-matched controls
GFAP: Glial fibrillary acidic protein
IL-1β: Interleukin-1β
MCI: Mild cognitive impairment
MMSE: Mini-Mental State Examination
p-Tau: Phospho-Tau
TNF-α: Tumor necrosis factor-alpha

## References

[1] Suescun J, Chandra S, Schiess MC, Actor JK, Smith KC (2019). Chapter 13 - The role of neuroinflammation in neurodegenerative disorders. Translational Inflammation.

[2] DiSabato DJ, Quan N, Godbout JP (2016). Neuroinflammation: the devil is in the details. J Neurochem.

[3] Lyman M, Lloyd DG, Ji X, Vizcaychipi MP, Ma D (2014). Neuroinflammation: the role and consequences. Neurosci Res.

[4] Mishra A, Kim HJ, Shin AH, Thayer SA (2012). Synapse loss induced by interleukin-1β requires pre- and post-synaptic mechanisms. J Neuroimmune Pharmacol.

[5] Subramanian J, Savage JC, Tremblay MÈ (2020). Synaptic loss in Alzheimer’s disease: mechanistic insights provided by two-photon in vivo imaging of transgenic mouse models. Front Cell Neurosci.

[6] Chen GF, Xu TH, Yan Y, Zhou YR, Jiang Y, Melcher K (2017). Amyloid-β (1–42) beta: structure, biology and structure-based therapeutic development. Acta Pharmacol Sin..

[7] Sun X, Chen WD, Wang YD (2015). β-Amyloid-β (1–42): the key peptide in the pathogenesis of Alzheimer’s DISEASE. Front Pharmacol.

[8] Hampel H, Hardy J, Blennow K, Chen C, Perry G, Kim SH (2021). The amyloid-β (1–42)-β pathway in Alzheimer’s disease. Mol Psychiatry.

[9] Knopman DS, Amieva H, Petersen RC, Chételat G, Holtzman DM, Hyman BT (2021). Alzheimer disease. Nat Rev Dis Primers.

[10] Gu L, Guo Z (2013). Alzheimer’s Aβ42 and Aβ40 peptides form interlaced Amyloid-β (1–42) fibrils. J Neurochem.

[11] Bettens K, Sleegers K, Van Broeckhoven C (2010). Current status on Alzheimer disease molecular genetics: from past, to present, to future. Hum Mol Genet.

[12] George-Hyslop PHS (2000). Molecular genetics of Alzheimer’s disease. Biol Psychiat.

[13] Li NM, Liu KF, Qiu YJ, Zhang HH, Nakanishi H, Qing H (2019). Mutations of beta-Amyloid-β (1–42) precursor protein alter the consequence of Alzheimer’s disease pathogenesis. Neural Regen Res.

[14] Shen J, Kelleher RJ (2007). The presenilin hypothesis of Alzheimer’s disease: evidence for a loss-of-function pathogenic mechanism. Proc Natl Acad Sci U S A.

[15] Liu M, Sui D, Dexheimer T, Hovde S, Deng X, Wang KW (2020). Hyperphosphorylation renders Tau prone to aggregate and to cause cell death. Mol Neurobiol.

[16] Ferrer I, Andrés-Benito P, Ausín K, Pamplona R, del Rio JA, Fernández-Irigoyen J (2021). Dysregulated protein phosphorylation: a determining condition in the continuum of brain aging and Alzheimer’s disease. Brain Pathol.

[17] Goel P, Chakrabarti S, Goel K, Bhutani K, Chopra T, Bali S (2022). Neuronal cell death mechanisms in Alzheimer’s disease: an insight. Front Mol Neurosci.

[18] Zhang W, Xiao D, Mao Q, Xia H (2023). Role of neuroinflammation in neurodegeneration development. Sig Transduct Target Ther.

[19] Choi SB, Kwon S, Kim JH, Ahn NH, Lee JH, Yang SH (2023). The molecular mechanisms of neuroinflammation in Alzheimer’s disease, the consequence of neural cell death. Int J Mol Sci.

[20] Rajesh Y, Kanneganti TD (2022). Innate immune cell death in neuroinflammation and Alzheimer’s disease. Cells.

[21] Skaper SD, Facci L, Zusso M, Giusti P (2018). An inflammation-centric view of neurological disease: beyond the neuron. Front Cell Neurosci.

[22] Brucato DR, Benjamin DE (2020). Synaptic pruning in Alzheimer’s disease: role of the complement system. Global Journal of Medical Research.

[23] Piccioni G, Mango D, Saidi A, Corbo M, Nisticò R (2021). Targeting microglia-synapse interactions in Alzheimer’s disease. Int J Mol Sci.

[24] Mori Y, Tsuji M, Oguchi T, Kasuga K, Kimura A, Futamura A (2021). Serum BDNF as a potential biomarker of Alzheimer’s disease: verification through assessment of serum, cerebrospinal fluid, and medial temporal lobe atrophy. Front Neurol.

[25] Giacomucci G, Mazzeo S, Bagnoli S, Ingannato A, Leccese D, Berti V (2022). Plasma neurofilament light chain as a biomarker of Alzheimer’s disease in subjective cognitive decline and mild cognitive impairment. J Neurol.

[26] Kim KY, Shin KY, Chang KA (2023). GFAP as a potential biomarker for Alzheimer’s disease: a systematic review and meta-analysis. Cells.

[27] Ally M, Sugarman MA, Zetterberg H, Blennow K, Ashton NJ, Karikari TK (2023). Cross-sectional and longitudinal evaluation of plasma glial fibrillary acidic protein to detect and predict clinical syndromes of Alzheimer’s disease. Alzheimers Dement (Amst).

[28] Utz J, Berner J, Muñoz LE, Oberstein TJ, Kornhuber J, Herrmann M (2021). Cerebrospinal fluid of patients with Alzheimer’s disease contains increased percentages of synaptophysin-bearing microvesicles. Front Aging Neurosci.

[29] Bruno D, Schurmann VS (2019). Addenbrooke’s cognitive examination III in the diagnosis of dementia: a critical review. Neuropsychiatr Dis Treat.

[30] Matías-Guiu JA, Valles-Salgado M, Rognoni T, Hamre-Gil F, Moreno-Ramos T, Matías-Guiu J (2017). Comparative diagnostic accuracy of the ACE-III, MIS, MMSE, MoCA, and RUDAS for screening of Alzheimer disease. Dement Geriatr Cogn Disord.

[31] Bajpai S, Upadhyay A, Sati H, Pandey RM, Chaterjee P, Dey AB (2020). Hindi version of Addenbrook’s Cognitive Examination III: distinguishing cognitive impairment among older Indians at the lower cut-offs. Clin Interv Aging.

[32] Howitt J, Hill AF (2016). Exosomes in the pathology of neurodegenerative diseases. J Biol Chem.

[33] Rastogi S, Sharma V, Bharti PS, Rani K, Modi GP, Nikolajeff F (2021). The evolving landscape of exosomes in neurodegenerative diseases: exosomes characteristics and a promising role in early diagnosis. Int J Mol Sci.

[34] Kalluri R, LeBleu VS (2020). The biology, function, and biomedical applications of exosomes. Science..

[35] Gomes P, Tzouanou F, Skolariki K, Vamvaka-Iakovou A, Noguera-Ortiz C, Tsirtsaki K (2022). Extracellular vesicles and Alzheimer’s disease in the novel era of Precision Medicine: implications for disease progression, diagnosis and treatment. Exp Neurol.

[36] Cocucci E, Meldolesi J (2015). Ectosomes and exosomes: shedding the confusion between extracellular vesicles. Trends Cell Biol.

[37] Pegtel DM, Gould SJ (2019). Exosomes. Annu Rev Biochem.

[38] Gámez-Valero A, Campdelacreu J, Vilas D, Ispierto L, Reñé R, Álvarez R (2019). Exploratory study on microRNA profiles from plasma-derived extracellular vesicles in Alzheimer’s disease and dementia with Lewy bodies. Transl Neurodegener.

[39] Lim WQ, Michelle Luk KH, Lee KY, Nurul N, Loh SJ, Yeow ZX (2023). Small extracellular vesicles’ miRNAs: biomarkers and therapeutics for neurodegenerative diseases. Pharmaceutics.

[40] Banks WA, Sharma P, Bullock KM, Hansen KM, Ludwig N, Whiteside TL (2020). Transport of extracellular vesicles across the blood-brain barrier: brain pharmacokinetics and effects of inflammation. Int J Mol Sci.

[41] Zhou F, Ebea P, Mutai E, Wang H, Sukreet S, Navazesh S (2022). Small extracellular vesicles in milk cross the blood-brain barrier in murine cerebral cortex endothelial cells and promote dendritic complexity in the hippocampus and brain function in C57BL/6J mice. Front Nutr.

[42] Eren E, Leoutsakos JM, Troncoso J, Lyketsos CG, Oh ES, Kapogiannis D (2022). Neuronal-derived EV biomarkers track cognitive decline in Alzheimer’s disease. Cells.

[43] Asai H, Ikezu S, Tsunoda S, Medalla M, Luebke J, Haydar T (2015). Depletion of microglia and inhibition of exosome synthesis halt tau propagation. Nat Neurosci.

[44] Söllvander S, Nikitidou E, Brolin R, Söderberg L, Sehlin D, Lannfelt L (2016). Accumulation of amyloid-β (1–42)-β by astrocytes result in enlarged endosomes and microvesicle-induced apoptosis of neurons. Mol Neurodegener.

[45] Sardar Sinha M, Ansell-Schultz A, Civitelli L, Hildesjö C, Larsson M, Lannfelt L (2018). Alzheimer’s disease pathology propagation by exosomes containing toxic amyloid-β (1–42)-beta oligomers. Acta Neuropathol.

[46] Zyśk M, Beretta C, Naia L, Dakhel A, Påvénius L, Brismar H (2023). Amyloid-β (1–42)-β accumulation in human astrocytes induces mitochondrial disruption and changed energy metabolism. J Neuroinflammation.

[47] Gabrielli M, Prada I, Joshi P, Falcicchia C, D’Arrigo G, Rutigliano G (2022). Microglial large extracellular vesicles propagate early synaptic dysfunction in Alzheimer’s disease. Brain.

[48] Beretta C, Nikitidou E, Streubel-Gallasch L, Ingelsson M, Sehlin D, Erlandsson A (2020). Extracellular vesicles from amyloid-β (1–42)-β exposed cell cultures induce severe dysfunction in cortical neurons. Sci Rep.

[49] Chen Y, He Y, Han J, Wei W, Chen F (2023). Blood-brain barrier dysfunction and Alzheimer’s disease: associations, pathogenic mechanisms, and therapeutic potential. Front Aging Neurosci.

[50] Garcia-Contreras M, Thakor AS (2022). Extracellular vesicles in Alzheimer’s disease: from pathology to therapeutic approaches. Neural Regen Res.

[51] Vella LJ, Hill AF, Cheng L (2016). Focus on extracellular vesicles: exosomes and their role in protein trafficking and biomarker potential in Alzheimer’s and Parkinson’s disease. Int J Mol Sci.

[52] Huo L, Du X, Li X, Liu S, Xu Y (2021). The emerging role of neural cell-derived exosomes in intercellular communication in health and neurodegenerative diseases. Front Neurosci.

[53] Rastogi S, Rani K, Rai S, Singh R, Bharti PS, Sharma V (2023). Fluorescence-tagged salivary small extracellular vesicles as a nanotool in early diagnosis of Parkinson’s disease. BMC Med.

[54] Rani K, Rastogi S, Vishwakarma P, Bharti PS, Sharma V, Renu K (2021). A novel approach to correlate the salivary exosomes and their protein cargo in the progression of cognitive impairment into Alzheimer’s disease. J Neurosci Methods.

[55] Rai S, Bharti PS, Singh R, Rastogi S, Rani K, Sharma V (2023). Circulating plasma miR-23b-3p as a biomarker target for idiopathic Parkinson’s disease: comparison with small extracellular vesicle miRNA. Front Neurosci.

[56] Théry C, Witwer KW, Aikawa E, Alcaraz MJ, Anderson JD, Andriantsitohaina R (2018). Minimal information for studies of extracellular vesicles 2018 (MISEV2018): a position statement of the International Society for Extracellular Vesicles and update of the MISEV2014 guidelines. J Extracell Vesicles.

[57] Ng A, Tam WW, Zhang MW, Ho CS, Husain SF, McIntyre RS (2018). IL-1β, IL-6, TNF- α and CRP in elderly patients with depression or Alzheimer’s disease: systematic review and meta-analysis. Sci Rep.

[58] Hulshof LA, van Nuijs D, Hol EM, Middeldorp J (2022). The role of astrocytes in synapse loss in Alzheimer’s disease: a systematic review. Front Cell Neurosci.

[59] Sze CI, Troncoso JC, Kawas C, Mouton P, Price DL, Martin LJ (1997). Loss of the presynaptic vesicle protein synaptophysin in hippocampus correlates with cognitive decline in Alzheimer disease. J Neuropathol Exp Neurol.

[60] Yuyama K, Igarashi Y (2017). Exosomes as carriers of Alzheimer’s amyloid-β (1–42)-ß. Front Neurosci.

[61] Watson LS, Hamlett ED, Stone TD, Sims-Robinson C (2019). Neuronally derived extracellular vesicles: an emerging tool for understanding Alzheimer’s disease. Mol Neurodegener.

[62] Gomes DE, Witwer KW (2022). L1CAM-associated extracellular vesicles: a systematic review of nomenclature, sources, separation, and characterization. J Extracell Biol.

[63] Goetzl EJ, Kapogiannis D, Schwartz JB, Lobach IV, Goetzl L, Abner EL (2016). Decreased synaptic proteins in neuronal exosomes of frontotemporal dementia and Alzheimer’s disease. FASEB J.

[64] Goetzl EJ, Abner EL, Jicha GA, Kapogiannis D, Schwartz JB (2018). Declining levels of functionally specialized synaptic proteins in plasma neuronal exosomes with progression of Alzheimer’s disease. FASEB J.

[65] Jung YJ, Tweedie D, Scerba MT, Greig NH (2019). Neuroinflammation as a factor of neurodegenerative disease: thalidomide analogs as treatments. Front Cell Develop Biol.

[66] Manolopoulos A, Delgado-Peraza F, Mustapic M, Pucha KA, Nogueras-Ortiz C, Daskalopoulos A (2023). Comparative assessment of Alzheimer’s disease-related biomarkers in plasma and neuron-derived extracellular vesicles: a nested case-control study. Front Mol Biosci.

[67] He Z, Guo JL, McBride JD, Narasimhan S, Kim H, Changolkar L (2018). Amyloid-β (1–42)-β plaques enhance Alzheimer’s brain tau-seeded pathologies by facilitating neuritic plaque tau aggregation. Nat Med.

[68] Pekeles H, Qureshi HY, Paudel HK, Schipper HM, Gornistky M, Chertkow H (2018). Development and validation of a salivary tau biomarker in Alzheimer’s disease. Alzheimers Dement (Amst)..

[69] Silva MC, Ferguson FM, Cai Q, Donovan KA, Nandi G, Patnaik D (2019). Targeted degradation of aberrant tau in frontotemporal dementia patient-derived neuronal cell models. Elife.

[70] Holper S, Watson R, Yassi N (2022). Tau as a biomarker of neurodegeneration. Int J Mol Sci.

[71] Lleó A, Núñez-Llaves R, Alcolea D, Chiva C, Balateu-Paños D, Colom-Cadena M (2019). Changes in synaptic proteins precede neurodegeneration markers in preclinical Alzheimer’s disease cerebrospinal fluid. Mol Cell Proteomics.

[72] Guo T, Zhang D, Zeng Y, Huang TY, Xu H, Zhao Y (2020). Molecular and cellular mechanisms underlying the pathogenesis of Alzheimer’s disease. Mol Neurodegener.

[73] Breijyeh Z, Karaman R (2020). Comprehensive review on Alzheimer’s disease: causes and treatment. Molecules.

[74] Snellman A, Ekblad LL, Koivumäki M, Lindgrén N, Tuisku J, Perälä M (2022). ASIC-E4: interplay of beta-amyloid-β (1–42), synaptic density and neuroinflammation in cognitively normal volunteers with three levels of genetic risk for late-onset Alzheimer’s disease – study protocol and baseline characteristics. Front Neurol.

[75] Goetzl EJ, Kapogiannis D, Schwartz JB, Lobach IV, Goetzl L, Abner EL (2016). Decreased synaptic proteins in neuronal exosomes of frontotemporal dementia and Alzheimer’s disease. FASEB J.

[76] Kinney JW, Bemiller SM, Murtishaw AS, Leisgang AM, Salazar AM, Lamb BT (2018). Inflammation as a central mechanism in Alzheimer’s disease. Alzheimers Dement (N Y).

[77] Mrak RE, Griffin WST (2000). Interleukin-1 and the immunogenetics of Alzheimer disease. J Neuropathol Exp Neurol.

[78] Frankola KA, Greig NH, Luo W, Tweedie D (2011). Targeting TNF-alpha to elucidate and ameliorate neuroinflammation in neurodegenerative diseases. CNS Neurol Disord Drug Targets.

[79] Jayaraman A, Htike TT, James R, Picon C, Reynolds R (2021). TNF-mediated neuroinflammation is linked to neuronal necroptosis in Alzheimer’s disease hippocampus. Acta Neuropathol Commun.

[80] Kim KY, Shin KY, Chang KA (2023). GFAP as a potential biomarker for Alzheimer’s disease: a systematic review and meta-analysis. Cells.

[81] Jain P, Wadhwa PK, Jadhav HR (2015). Reactive astrogliosis: role in Alzheimer’s disease. CNS Neurol Disord Drug Targets.

[82] Kamphuis W, Mamber C, Moeton M, Kooijman L, Sluijs JA, Jansen AHP (2012). GFAP isoforms in adult mouse brain with a focus on neurogenic astrocytes and reactive astrogliosis in mouse models of Alzheimer disease. PLoS ONE.

[83] Chatterjee P, Pedrini S, Stoops E, Goozee K, Villemagne VL, Asih PR (2021). Plasma glial fibrillary acidic protein is elevated in cognitively normal older adults at risk of Alzheimer’s disease. Transl Psychiatry.

[84] Kalluri R, LeBleu VS (2020). The biology, function, and biomedical applications of exosomes. Science.

