# Supplementary material for: Circulating small extracellular vesicles in Alzheimer’s disease: a case–control study of neuro-inflammation and synaptic dysfunction
Source: BMC Med. 2024 Jun 20;22:254. doi: 10.1186/s12916-024-03475-z (PMC11188177; doi:10.1186/s12916-024-03475-z)
Supplement: Supplementary file 1 — Additional file 1: Fig S1. [CD63 expression in age-matched controls (AMC), mild cognitive impairment (MCI), and Alzheimer’s disease (AD) and their densitometric analysis]. Fig S2. [CD81 expression in age-matched controls (AMC), mild cognitive impairment (MCI), and Alzheimer’s disease (AD) and their densitometric analysis]. Fig S3. [TSG101 expression in age-matched controls (AMC), mild cognitive impairment (MCI), and Alzheimer’s disease (AD) and their densitometric analysis]. Fig S4. [L1CAM expression in age-matched controls (AMC), mild cognitive impairment (MCI), and Alzheimer’s disease (AD) and their densitometric analysis]. Fig S5. [Synaptophysin (SYP) expression in age-matched controls (AMC), mild cognitive impairment (MCI), and Alzheimer’s disease (AD) and their densitometric analysis]. Fig S6. [Glial Fibrillary Acidic Protein (GFAP) expression in age-matched controls (AMC), mild cognitive impairment (MCI), and Alzheimer’s disease (AD) and their densitometric analysis]. Fig S7. [β-Actin expression in age-matched controls (AMC), mild cognitive impairment (MCI), and Alzheimer’s disease (AD) and their densitometric analysis]. Fig S8. [Amyloidβ-42 Oligomer expression in age-matched controls (AMC), mild cognitive impairment (MCI), and Alzheimer’s disease (AD) and their densitometric analysis]. Fig S9. [IL1β (A) and TNFα (B) expression in age-matched controls (AMC), mild cognitive impairment (MCI), and Alzheimer’s disease (AD) and their densitometric analysis]. Fig S10. [p-Tau expression in age-matched controls (AMC), mild cognitive impairment (MCI), and Alzheimer’s disease (AD) and their densitometric analysis]. [file 12916_2024_3475_MOESM1_ESM.docx]

**ADDITIONAL FILE 1 (Fig. 1-10)**


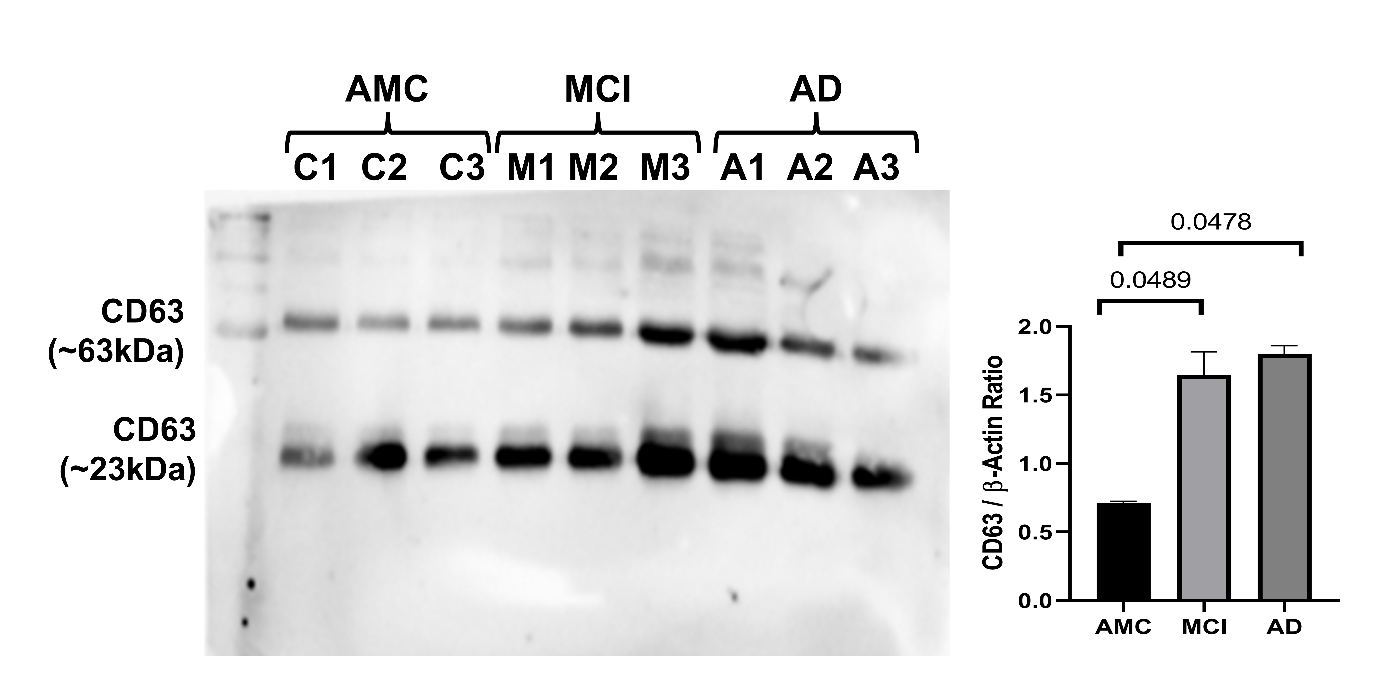


**Figure S1:** CD63 expression in age-matched controls (AMC), mild cognitive impairment (MCI), and Alzheimer’s disease (AD) and their densitometric analysis.


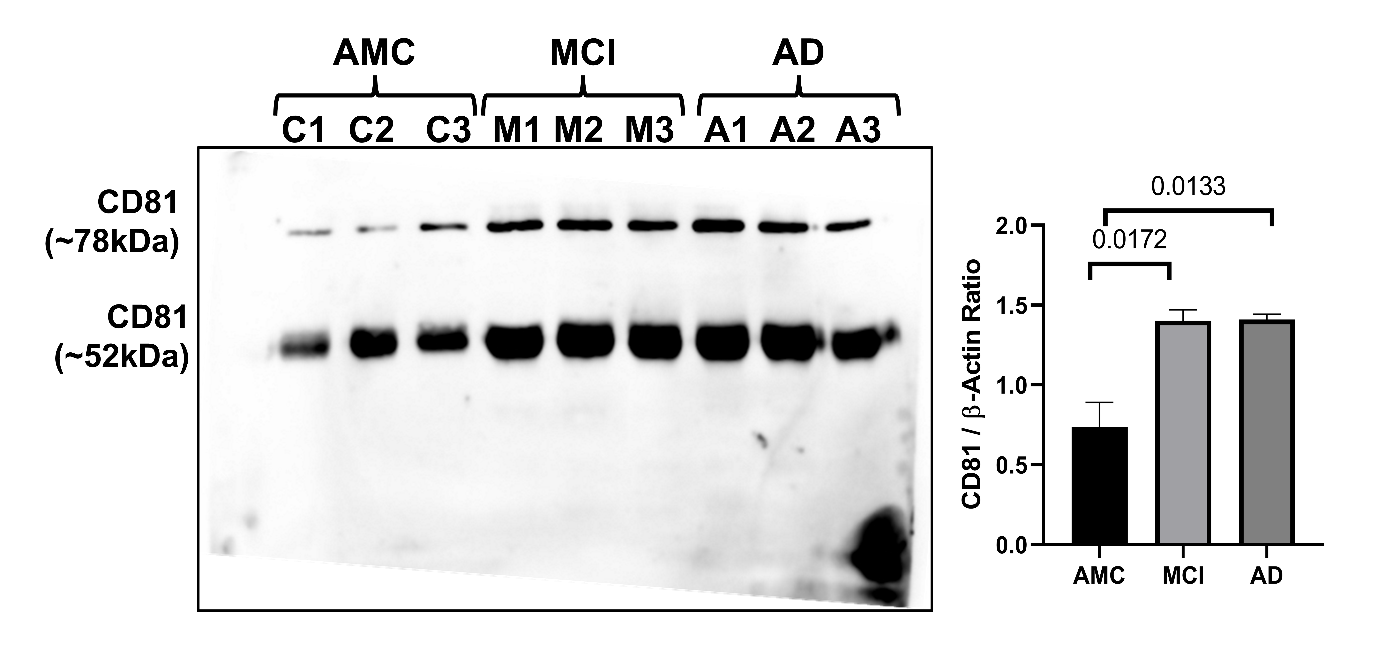


**Figure S2:** CD81 expression in age-matched controls (AMC), mild cognitive impairment (MCI), and Alzheimer’s disease (AD) and their densitometric analysis.


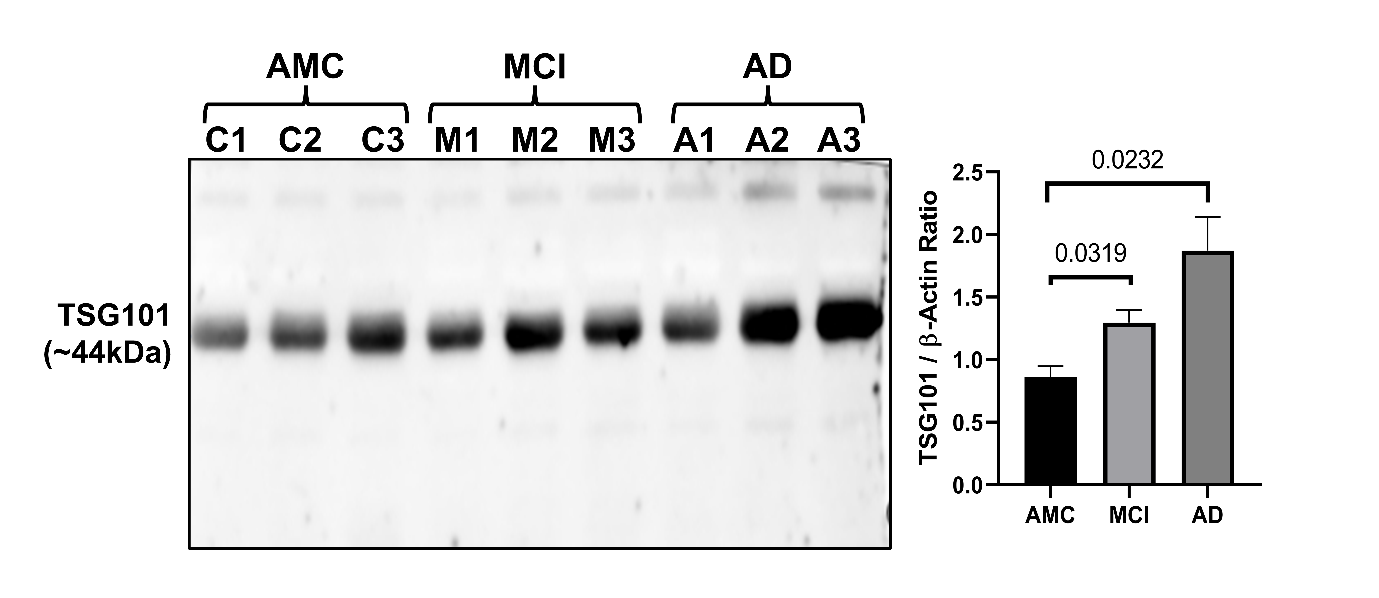


**Figure S3:** TSG101 expression in age-matched controls (AMC), mild cognitive impairment (MCI), and Alzheimer’s disease (AD) and their densitometric analysis.


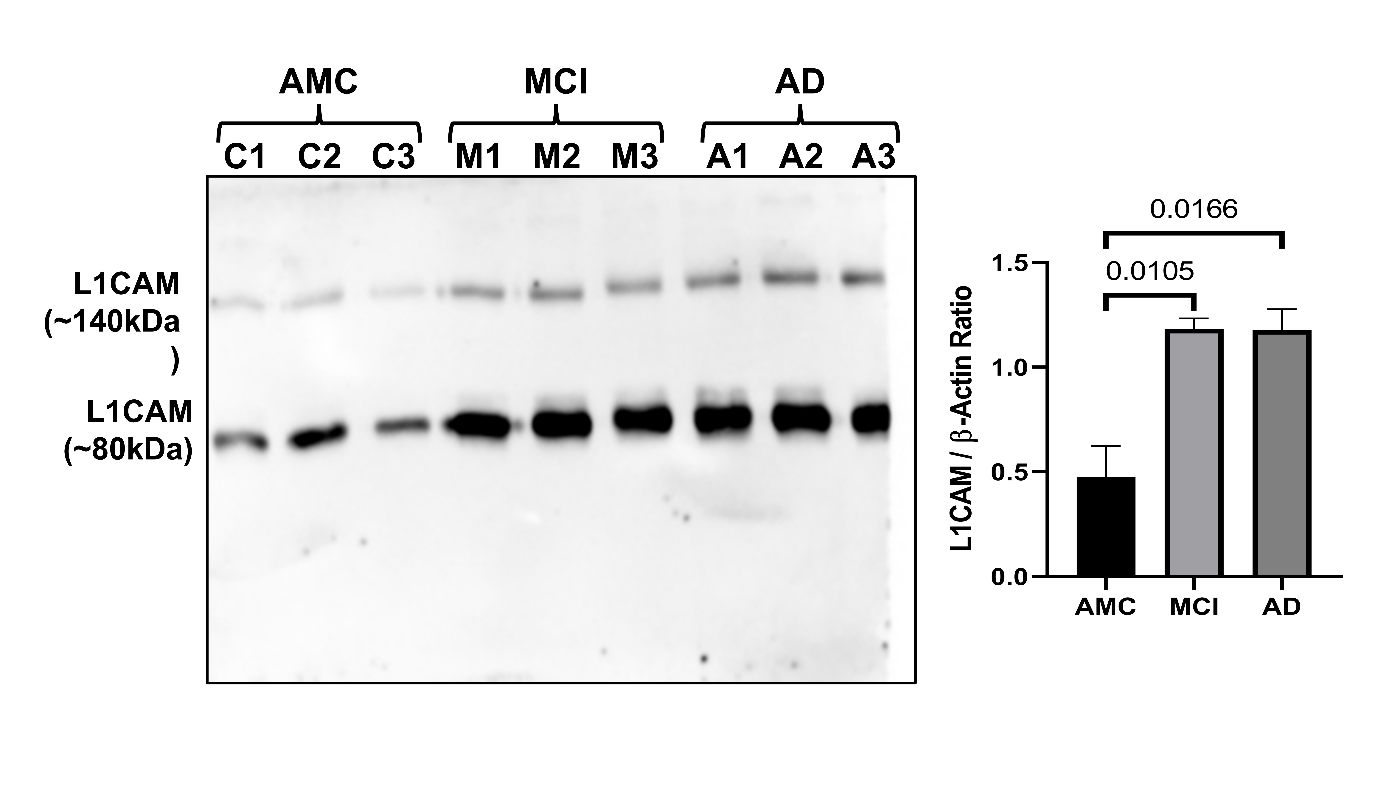


**Figure S4:** L1CAM expression in age-matched controls (AMC), mild cognitive impairment (MCI), and Alzheimer’s disease (AD) and their densitometric analysis.


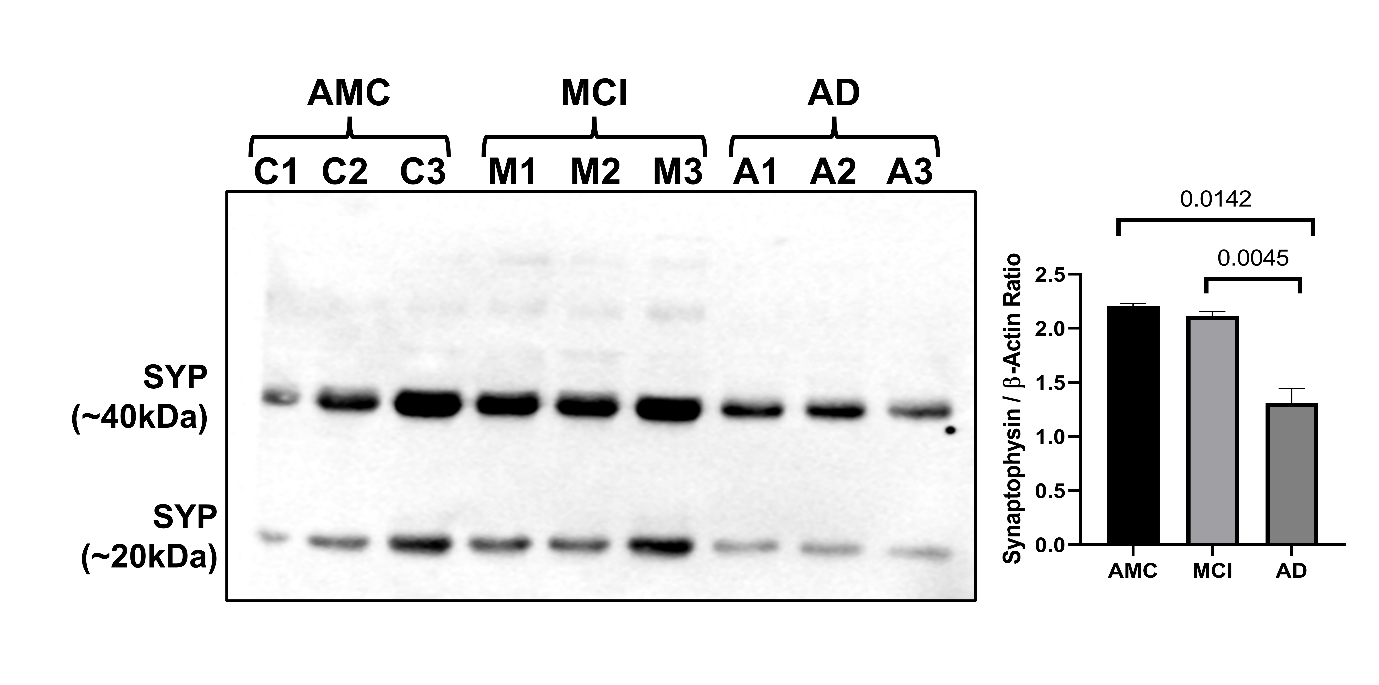


**Figure S5:** Synaptophysin (SYP) expression in age-matched controls (AMC), mild cognitive impairment (MCI), and Alzheimer’s disease (AD) and their densitometric analysis.


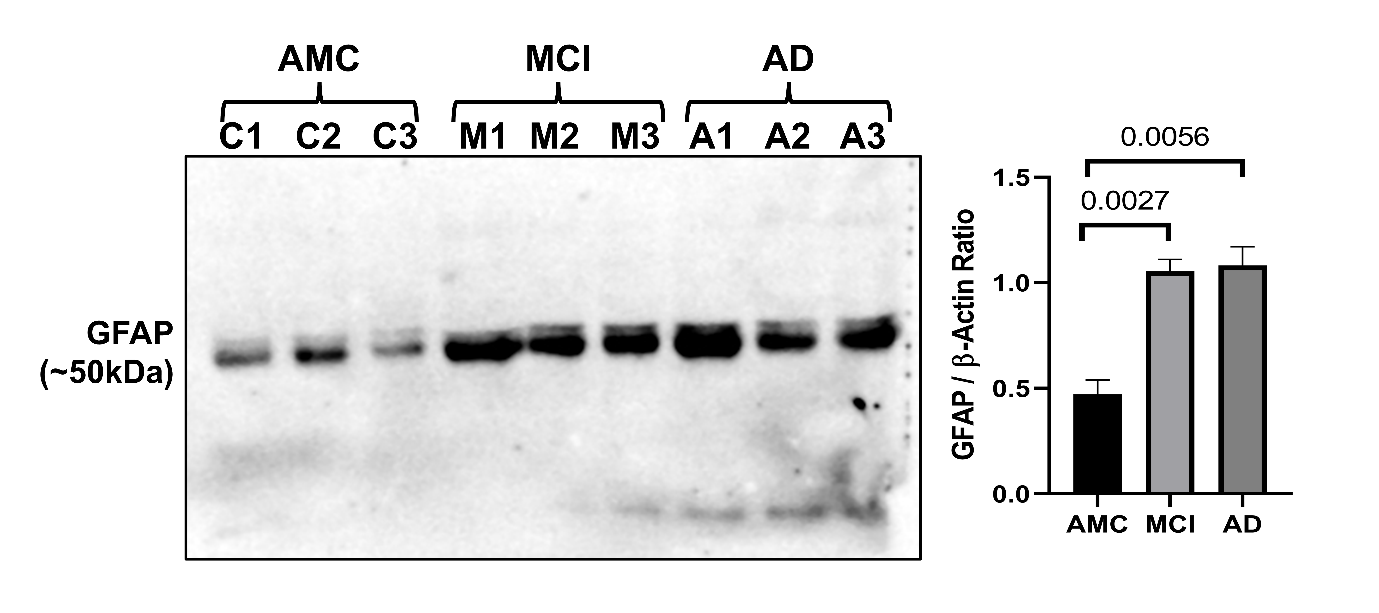


**Figure S6:** Glial Fibrillary Acidic Protein (GFAP) expression in age-matched controls (AMC), mild cognitive impairment (MCI), and Alzheimer’s disease (AD) and their densitometric analysis.


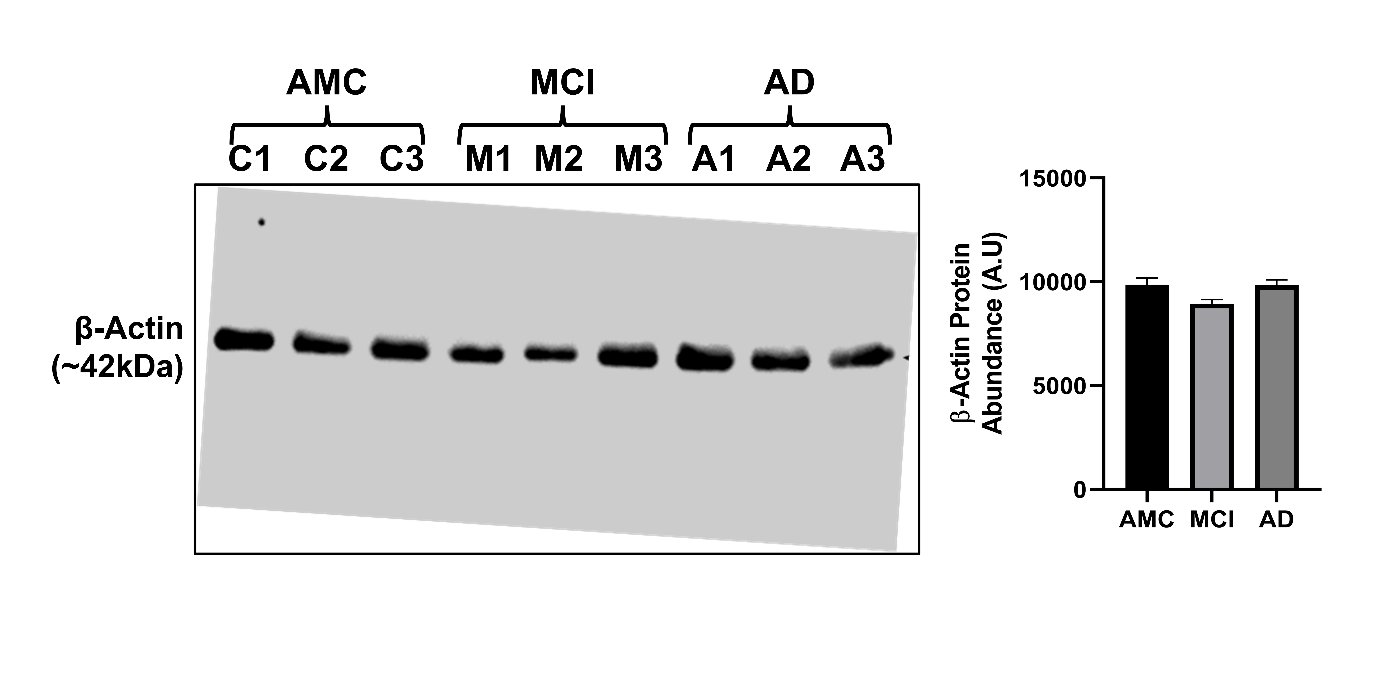


**Figure S7:** β-Actin expression in age-matched controls (AMC), mild cognitive impairment (MCI), and Alzheimer’s disease (AD) and their densitometric analysis.


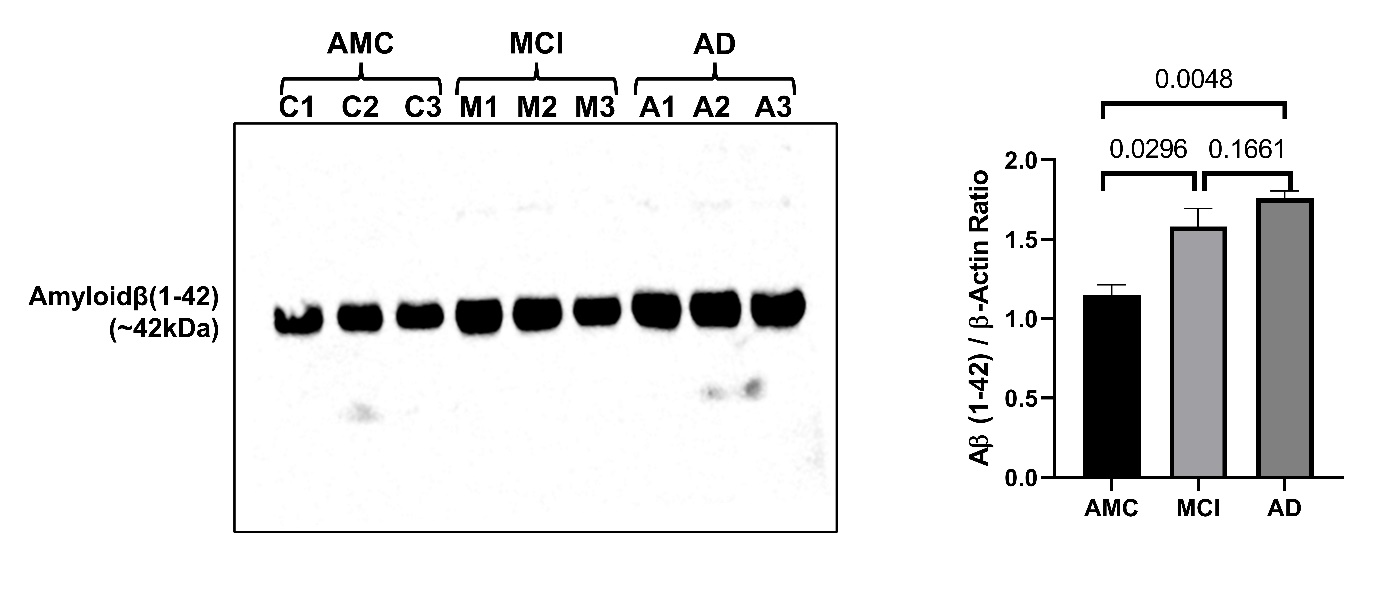


**Figure S8:** Amyloidβ-42 Oligomer expression in age-matched controls (AMC), mild cognitive impairment (MCI), and Alzheimer’s disease (AD) and their densitometric analysis.


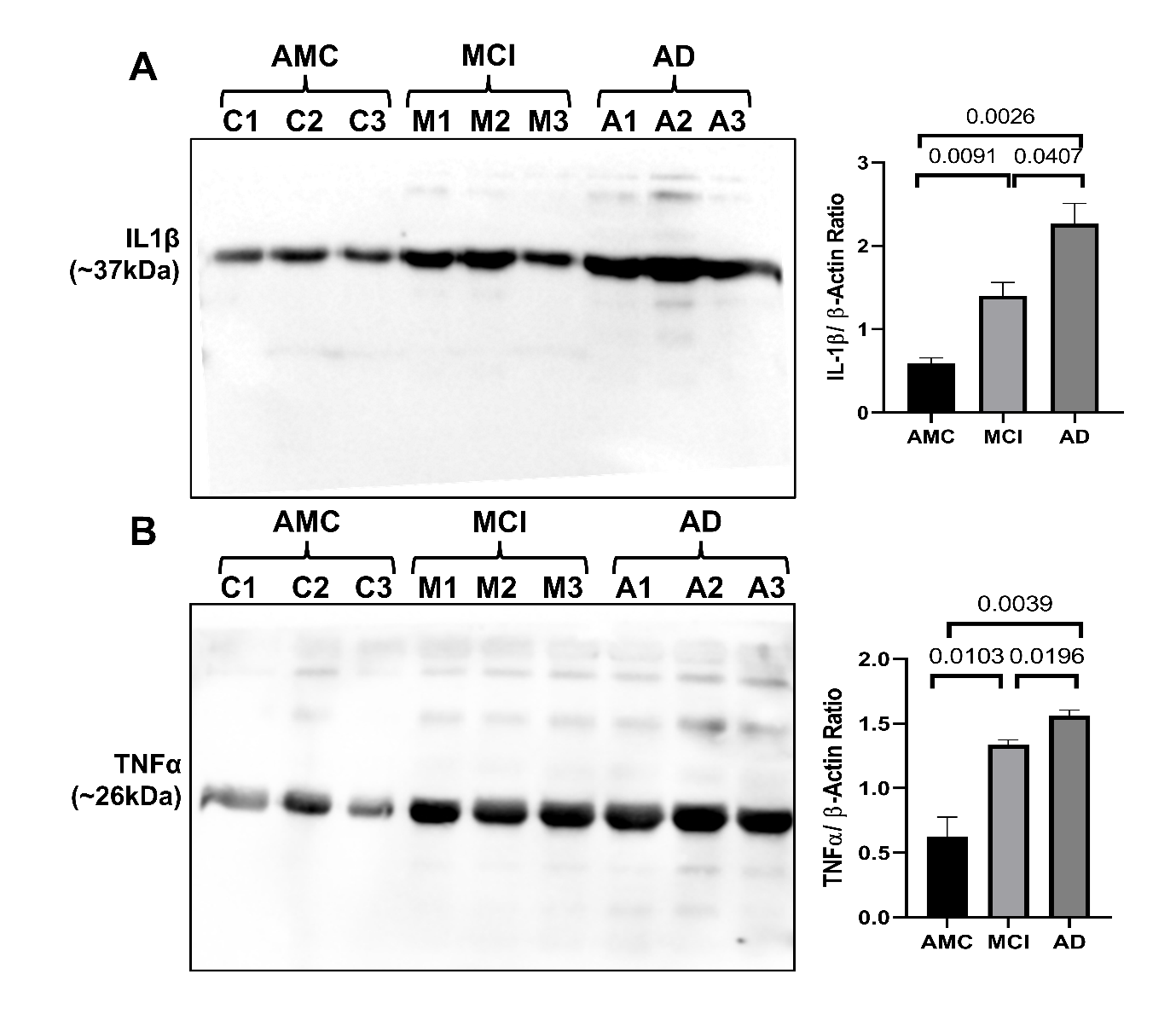
**Figure S9:** IL1β (A) and TNFα (B) expression in age-matched controls (AMC), mild cognitive impairment (MCI), and Alzheimer’s disease (AD) and their densitometric analysis.


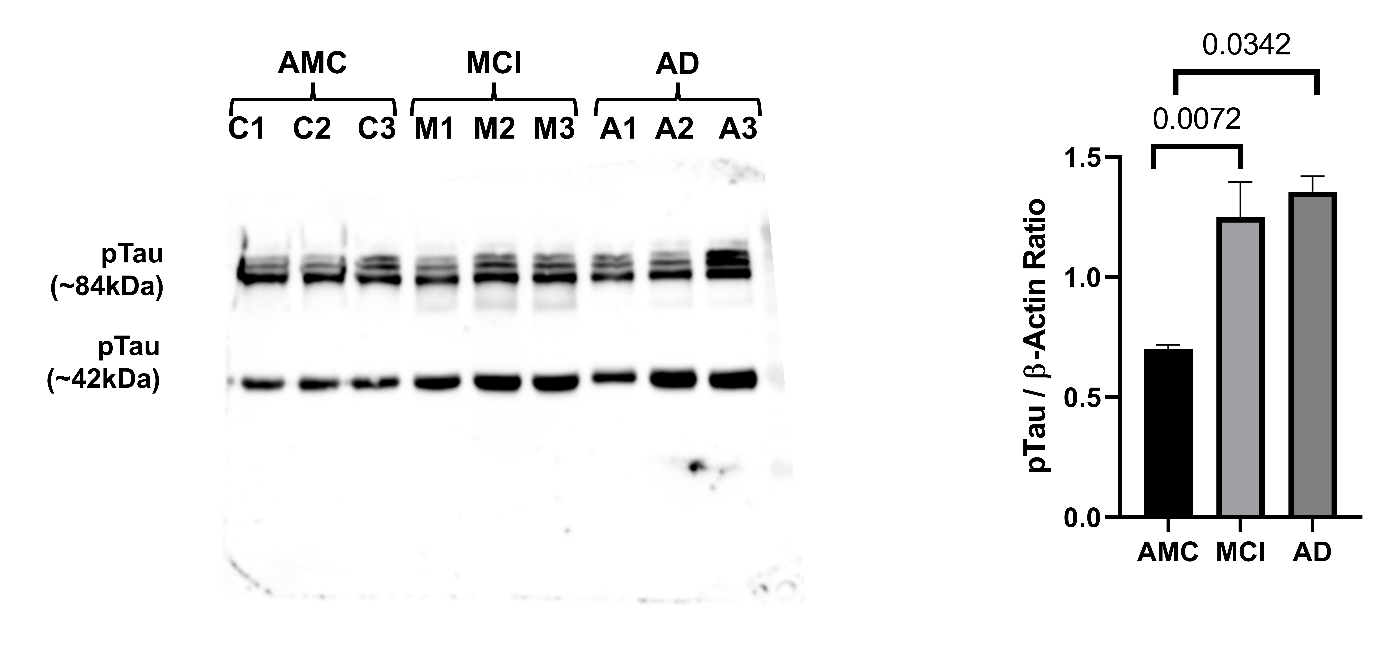


**Figure S10:** p-Tau expression in age-matched controls (AMC), mild cognitive impairment (MCI), and Alzheimer’s disease (AD) and their densitometric analysis
